# Isoprene enhances leaf cytokinin metabolism and induces early senescence

**DOI:** 10.1111/nph.17833

**Published:** 2021-12-20

**Authors:** Kaidala Ganesha Srikanta Dani, Susanna Pollastri, Sara Pinosio, Michael Reichelt, Thomas D. Sharkey, Jörg‐Peter Schnitzler, Francesco Loreto

**Affiliations:** ^1^ Institute for Sustainable Plant Protection National Research Council of Italy Via Madonna del Piano 10 50019 Sesto Fiorentino Florence Italy; ^2^ Department of Biology, Agriculture and Food Sciences National Research Council of Italy Piazzale Aldo Moro 7 00185 Rome Italy; ^3^ Institute of Biosciences and Bioresources National Research Council of Italy Via Madonna del Piano 10 50019 Sesto Fiorentino Florence Italy; ^4^ Institute for Applied Genomics Via Jacopo Linussio 51 33100 Udine Italy; ^5^ Department of Biochemistry Max Planck Institute for Chemical Ecology Hans‐Knöll Strasse 8 D‐07745 Jena Germany; ^6^ MSU‐DOE Plant Research Laboratory Department of Biochemistry and Molecular Biology Michigan State University East Lansing MI 48824 USA; ^7^ Research Unit Environmental Simulation Institute of Biochemical Plant Pathology Helmholtz Zentrum München German Research Center for Environmental Health 85764 Neuherberg Germany; ^8^ Department of Biology University of Naples Federico II Via Cinthia 80126 Naples Italy

**Keywords:** biogenic volatiles, cytokinins, hormones, isoprene, leaf senescence, signalling, transcriptomics, unstressed plants

## Abstract

- Isoprene, a major biogenic volatile hydrocarbon of climate‐relevance, indisputably mitigates abiotic stresses in emitting plants. However functional relevance of constitutive isoprene emission in unstressed plants remains contested. Isoprene and cytokinins (CKs) are synthesized from a common substrate and pathway in chloroplasts. It was postulated that isoprene emission may affect CK‐metabolism.
- Using transgenic isoprene‐emitting (IE) *Arabidopsis* and isoprene nonemitting (NE) RNA‐interference grey poplars (paired with respective NE and IE genotypes), the life of individual IE and NE leaves from emergence to abscission was followed under stress‐free conditions. We monitored plant growth rate, aboveground developmental phenotype, modelled leaf photosynthetic energy status, quantified the abundance of leaf CKs, analysed *Arabidopsis* and poplar leaf transcriptomes by RNA‐sequencing in presence and absence of isoprene during leaf senescence.
- Isoprene emission by unstressed leaves enhanced the abundance of CKs (isopentenyl adenine and its precursor) by > 200%, significantly upregulated genes coding for CK‐synthesis, CK‐signalling and CK‐degradation, hastened plant development, increased chloroplast metabolic rate, altered photosynthetic energy status, induced early leaf senescence in both *Arabidopsis* and poplar. IE leaves senesced sooner even in decapitated poplars where source–sink relationships and hormone homeostasis were perturbed.
- Constitutive isoprene emission significantly accelerates CK‐led leaf and organismal development and induces early senescence independent of growth constraints. Isoprene emission provides an early‐riser evolutionary advantage and shortens lifecycle duration to assist rapid diversification in unstressed emitters.

## Introduction

Isoprene (C_5_H_8_) is the most prominent biogenic volatile organic compound released by the leaves of some of the fastest‐growing and most speciose forest trees (Dani *et al*., 2014a). While isoprene in the atmosphere influences oxidation chemistry, secondary aerosol formation and global radiation budget (McFiggans *et al*., 2019; Lamkaddam *et al*., 2021), isoprene also serves emitting plants by mitigating transient heat and oxidative stresses (Behnke *et al*., 2007; Velikova *et al*., 2011; Pollastri *et al*., 2019). Isoprene emission and stress hormones are sensitive to each other and their interactions mediate plant stress responses (Behnke *et al*., 2010; Mutanda *et al*., 2016; Parveen *et al*., 2019; Xu *et al*., 2020). Overexpression or suppression of isoprene synthase and isoprene fumigation can modulate stress‐responsive gene expression (Harvey & Sharkey, 2016; Zuo *et al*., 2019), although the interactive modes are unclear. Carbon as CO_2_ is not a limiting factor for plant growth. Yet, any carbon that is fixed by photosynthesis but not converted into organic compounds (e.g. sugars) under optimal growth conditions is an expensive loss especially for perennials that endure stress and long periods of suboptimal photosynthesis. Isoprene is essentially a ‘loss of carbon’ fixed by photosynthesis (1–2% when unstressed, up to 10% if stressed; Peñuelas & Munné‐Bosch, 2005). Thus, it remains unclear why isoprene emission in light occurs continuously in unstressed leaves over their entire lifespan while recent evidence shows continuous emission even in dark‐acclimated unstressed heterotrophic algae (Dani *et al*., 2020). All of these suggest fundamental yet unknown house‐keeping functions for constitutively emitted isoprene.

Isoprene is made via the plastid‐localized methylerythritol phosphate (MEP) pathway by sourcing carbon and energy mainly from photosynthesis. The extended isoprenoid MEP pathway also makes several vital plant hormones (cytokinins, CKs; abscisic acid, ABA; strigolactones, SLs), and accessory photosynthetic and photoprotective pigments (β‐carotene and xanthophylls), all directly involved in regulating leaf senescence and lifespan (Dani *et al*., 2016). In particular, isoprene and CKs are synthesized concurrently from the substrate dimethylallyl diphosphate (DMADP). Adenosine triphosphate (ATP or its dephosphorylated states such as ADP and AMP) combines with DMADP in presence of isopentenyl transferase to give iP (isopentenyladenine)‐type CKs, whereas DMADP is directly converted to isoprene by isoprene synthase (Kasahara *et al*., 2004; Schnitzler *et al*., 2005). CKs, regarded as plant ‘youth hormones’, regulate leaf development and expansion, prevent chlorophyll degradation and keep leaves green, maintain chloroplast integrity, and mediate abiotic stress responses (Werner *et al*., 2001; Zavaleta‐Mancera *et al*., 2007; Nishiyama *et al*., 2011; Raines *et al*., 2016).

Given the metabolic proximity and potentially common constraints on the synthesis and functions of isoprene and iP‐type CKs, we tested if isoprene affects the course of natural leaf and plant senescence by influencing CK metabolism. We used two verified model plant systems namely (a) the model herbaceous annual *Arabidopsis thaliana* that naturally does not emit isoprene (nonemitting or NE), and *Arabidopsis* genetically transformed to emit isoprene (isoprene‐emitting or IE), and (b) the model perennial tree and naturally IE grey poplar (*Populus × canescens*), and grey poplars where isoprene synthesis and emission was suppressed by RNA‐interference or RNA*i* to obtain NE lines. We comprehensively analysed the impact of constitutive isoprene emission on plant developmental phenotypes and tracked the life of individual IE and NE leaves from emergence to abscission. We measured photosynthesis, modelled photosynthetic energy status of leaves, quantified the changes in the abundance of leaf CKs, and analysed the poplar and *Arabidopsis* transcriptome by RNA‐sequencing in presence and absence of isoprene during leaf senescence.

## Materials and Methods

### Plant material

#### Arabidopsis

*Arabidopsis thaliana* (*At*) Col‐0 ecotype was transformed to obtain IE lines using *Agrobacterium* and the floral dip method at the plant transformation facility in Michigan State University (East Lansing, MI, USA). The cloning construct included the complete coding DNA sequence (CDS) of isoprene synthase (*ISPS*) from *Eucalyptus globulus* downstream to the *Arabidopsis* Rubisco SSU promoter *rbcS‐1A*. Another construct lacking *ISPS* was used as the empty vector control. Seven independent transgenic lines were obtained (selected on kanamycin) until F3 transgenic seeds were obtained and verified by PCR, and deposited at the Arabidopsis Biological Resource Centre (ABRC) in Ohio State University, Columbus, OH, USA (Zuo *et al*., 2019). Seeds of *At* wild‐type (WT) control (Col‐0), empty vector control (line EV‐B3), and two IE lines (ISPS‐B2 and ISPS‐C4) were formally obtained from the ABRC and at that time we only knew that ISPS‐B2 and ISPS‐C4 lines emitted isoprene. The developmental phenotype and isoprene emission rates were enumerated independently at the National Research Council (CNR) research area in Florence (Italy). Once in Italy new seed stocks were generated. Seeds were surface sterilized in 70% ethanol, transferred to Petri plates containing Murashige and Skoog’s agar medium, and vernalized at 4°C for 48 h. Plates were then transferred to a growth cabinet and allowed to germinate at 18 ± 2°C, long days (16 h : 8 h, light : dark), light intensity of 100 µmol m^−2^ s^−1^ from white fluorescent tubes, and 40% relative humidity. Seedlings were individually transplanted to soil‐substrate containers placed in plastic trays and watered regularly. Inflorescence and pods were allowed to dry naturally while attached to the plants, and seeds were harvested. An independent set of plants of the four lines was grown under a light intensity of 200 µmol m^−2^ s^−1^, constituting high‐light treatment, keeping all other conditions identical to those used during seed germination.

#### Poplar

Isoprene‐emitting WT grey poplars (*Populus × canescens*) along with two transgenic lines in which expression of isoprene synthase (*PcISPS*) was suppressed by RNA*i* were used. Transgenics were generated and micro‐propagated at the Institute of Biochemical Plant Pathology, Helmholtz Centre in Munich (Germany). RNA*i*‐mediated post‐transcriptional silencing of isoprene synthase in poplars was enabled by the introduction of sense and antisense hairpin sequences (160 bp, highly specific to *ISPS*) in a binary vector via *Agrobacterium* mediated transformation (35S: PcISPS‐RNA*i*) (Behnke *et al*., 2007). Rooted 3‐month‐old cuttings (15 individuals each of isoprene‐emitting WT and empty vector (EV) lines, isoprene‐suppressed RA1 and RA2 lines) were brought to the CNR research area. The saplings were initially grown in 2 l pots containing soil substrate (25%), silica sand (25%), perlite (50% v/v) and slow‐release fertilizer. Young saplings were soon transplanted to 7 l pots (for 2 months) and later transplanted to 18 l pots with the same soil substrate with slow‐release fertilizer. Poplars were watered regularly and acclimated to natural seasonal variation in sun light intensity, photoperiod, temperature, and humidity in a CNR experimental facility for genetically modified organisms. After the first season (from April 2018 until December 2018), the main stems were pruned to get stubs (1.5 m) without any leaves or branches. These rooted‐stem cuttings from year 1 were transferred to 40 l pots in February of year 2. Budbreak commenced in March, and the second seasonal monitoring of leaf development and senescence went on until December 2019. The microclimate data in the experimental site for the two year of the experiment were gathered from the weather monitoring and modelling centre maintained by Consorzio LaMMA of CNR.

### Plant developmental phenotyping

#### Arabidopsis

The day *Arabidopsis* seeds germinated was noted day 0. Leaf samples were collected at six time points during the plant’s lifecycle, classified on the basis of days after germination (DAG) and flowering. Leaves were numbered according to their order of emergence from base to apex, excluding cotyledon leaves. Leaves in position 7, 8, 9, and 10 from the base (the biggest leaves) were marked. A first batch of plants was grown under the same light intensity at which plants germinated (100 µmol photons m^−2^ s^−1^). For this batch, leaves were sampled at 28 DAG (youngest stage sampled), 36 DAG (leaves 7 and 8 fully expanded), 48 DAG (fully mature plant body, prior to bolting), 56 DAG (early‐senescence phase, inflorescence seen in all four lines), 64 DAG (mid‐senescence phase), and finally at 76 DAG (late‐senescence phase, near‐end of lifecycle). Inflorescence was cut and weighed at 56, 64 and 76 DAG. A second batch of *Arabidopsis* plants were grown under 200 µmol photons m^−2^ s^−1^, and leaves in positions 7, 8, 9, and 10 were sampled at 24, 28, 36, and 48 DAG, respectively.

#### Poplar

Poplar leaves were tagged at the time of emergence. Leaves were grouped into three categories. (1) Spring leaves (emerging in May that became lower nodal leaves of the main stem), (2) Summer leaves (emerging in July that became intermediate nodal leaves of the stem), and (3) Autumn leaves (emerging in September, representing mature leaves near the stem apex). Leaf area was measured using a LI‐3000 portable area meter (Li‐Cor Biosciences Inc., Lincoln, NE, USA). Apical extension was measured once every fortnight to once a month, and sub‐seasonal trends in apical growth rate were calculated. At the end of the experiment (December), branching pattern was quantified by marking the branches (from base to apex) and by measuring their length and fresh weight post‐harvest.

#### Poplar ‘no‐growth’ scenarios

Plants were allowed to grow for one full growing season from April to October, and left undisturbed during natural senescence until December. One‐year‐old plants were completely pruned (1.5 m from the soil) in the following January, and repotted with new soil in March when they started sprouting. Five plants per each poplar line grew for the entire second growing season without pruning (from April to October, left undisturbed until December). Ten leaves per individual that emerged on the main stem within a span of 10 d in spring (June) were tagged. Another set of six to 10 leaves that emerged on the main stem in summer (July) were tagged on the day of emergence for photosynthesis measurements. Ten plants per poplar line grew until middle of July when the apex was cut. Decapitated poplars held onto 15–20 fully expanded leaves, whose date of emergence was known and those leaves were 45 ± 10 day old at the time of decapitation. Any new sprouting was removed within a couple of days after emergence. No new branches were allowed to grow for the remaining part of the year.

It is acknowledged that a significant proportion of nutrient recycled from senescing leaves can be supplied to roots and overwintering subterranean storage tissues in many perennial species (Scarascia‐Mugnozza *et al*., 1999; Byne & Ryser, 2020). We did not quantify belowground changes in this study. It is also noted that seasonal senescence in young poplar saplings may appear qualitatively different from typical autumn senescence in adult trees, although both are essentially the same processes, showing high variability among leaves within individuals (Keskitalo *et al*., 2005; Mattila *et al*., 2018), and both are driven by sink constraints and environmental cues. All poplars were grown in nutrient‐replete pots. The pots had enough soil volume (avoiding root limitation) and slow‐release fertilizers to support growth for 8 months (avoiding nitrogen deprivation). They were watered adequately throughout the experiment.

### Measurements of gas exchange and calculation of energy status and kinetic parameters

#### Arabidopsis

Net photosynthesis (*P*_n_), stomatal conductance (*g*_s_), and intercellular CO_2_ concentration (*C*_i_) were measured on fully expanded leaves in positions 8–12 (*n* = 5 individual plants) before flowering (40–48 DAG) and after flowering (64–72 DAG). Measurements were made between 11:00 h and 15:00 h, using a Li‐Cor 6400XT infrared gas analyser (Li‐Cor Biosciences). Leaves clamped in the circular leaf cuvette (area: 2 cm^2^) were maintained at 20°C, light intensity was set to 150 µmol m^−2^ s^−1^ for low‐light acclimated plants and 250 µmol m^−2^ s^−1^ for high‐light acclimated plants, volatile‐free clean air was humidified to achieve *c*. 40% relative humidity (leaf to air vapour pressure deficit was 0.9–1.2 kPa) and CO_2_ concentration was 400 µmol mol^−1^.

#### Poplar

In poplar leaves, all gas exchange measurements were carried out using a Li‐Cor 6400XT between 10:00 h and 15:00 h, on bright sunny days, and on individual mature leaves at three to four stages of a leaf lifecycle (see earlier). Leaf temperature was set to 24 ± 1°C in April, 28 ± 1°C in June/July, 24 ± 1°C in September/October, and 20 ± 1°C in November/December. In all measurements the relative humidity was maintained between 40% and 65%, ambient CO_2_ concentration was 400 µmol mol^−1^, and the light intensity was set to 1500 µmol m^−2^ s^−1^ (except for leaves senescing in November/December, when light intensity was set to 1000 µmol m^−2^ s^−1^).

Photosynthesis response to increases and decreases in CO_2_ concentrations (40–1600 µmol mol^−1^) was recorded in poplar leaves throughout their lifecycle. The *V*_cmax_ (maximum carboxylation rate of Rubisco) and *J* (instantaneous electron transport rate) were estimated by fitting net CO_2_ assimilation rate or *P*_n_ vs *C*_i_ curves using an excel based curve‐fitting tool (Sharkey, 2016). Isoprene synthesis is a minor yet important nonphotosynthetic and nonphotorespiratory energy sink within chloroplasts (Dani *et al*., 2014b). Hence, the chloroplast energy status of the leaves was quantified using the following equations:

(Eqn 1) $$R_{l}=\frac{1}{12}\left[J‐4\left(P_{n}+R_{d}\right)\right]$$

(Eqn 2) $$J_{c}=\frac{1}{3}\left[J+8\left(P_{n}+R_{d}\right)\right]$$

(Eqn 3) $$J_{o}=\frac{2}{3}\left[J‐4\left(P_{n}+R_{d}\right)\right]$$

(Eqn 4) $$v_{o}=\frac{1}{6}\left[J‐\frac{2}{3}\left(P_{n}+R_{d}\right)\right]$$

(Eqn 5) $$v_{c}=\left(P_{n}+R_{d}\right)+\frac{1}{2}v_{o}$$

where *P*_n_, net rate of CO_2_ uptake per unit of projected leaf area (μmol m^−2^ s^−1^); *C*_i_, intercellular CO_2_ concentration (μmol mol^−1^); *J*, instantaneous electron transport rate (μmol e^−^ m^−2^ s^−1^); *J*_c_, proportion of *J* utilized for carboxylation of RuBP by Rubisco; *J*_o_, proportion of *J* utilized for oxygenation of RuBP by Rubisco (photorespiration); *K*_m_, effective Michaelis–Menten coefficient for carboxylation by Rubisco (at 25°C); *R*_d_, day mitochondrial respiration rate (μmol m^−2^ s^−1^); *R*_l_, photorespiration rate (μmol m^−2^ s^−1^); *V*_cmax_, maximum rate of RuBP carboxylation by Rubisco (μmol m^−2^ s^−1^); *v*_c_, rate of carboxylation by Rubisco (μmol m^−2^ s^−1^); *v*_o_, rate of oxygenation by Rubisco (μmol m^−2^ s^−1^).

### Measurements of imaging chlorophyll fluorescence

The maximal quantum yield of chlorophyll fluorescence and photochemical efficiency of photosystem II (*F*_v_/*F*_m_) and the electron transport rate were estimated by saturating‐pulse chlorophyll fluorescence imaging using a Walz Imaging PAM (Heinz Walz, Effeltrich, Germany). For *Arabidopsis* grown in low‐light (100 µmol m^−2^ s^−1^) whole plants (< 28 DAG) and leaves cut from fully mature plants (> 42 DAG, except WT line) were used during imaging (*n* = 6 whole plants, or individual cut leaves harvested from four individuals). In poplars, individual cut leaves (two leaves of same age from *n* = 3 individuals) were sampled at 30, 60, 90, 120, and 180 d after emergence (DAE) and used for fluorescence measurements. All leaves were dark‐acclimated for 30 min before imaging.

### Isoprene sampling and quantification

Isoprene sampling was done from fully expanded leaves. In *Arabidopsis*, one or two leaves per individual (*n* = 4 individual plants per line), and in poplars, two to three mature leaves (leaf age between 30 to 60 d) per individual per line were sampled (*n* = 4–5 individual plants per line). A portion (300 ml min^−1^) of the Li‐Cor cuvette outflow was diverted using a mass flow pump (AP Buck Inc., Orlando, FL, USA) onto a cartridge filled with adsorbents (30 mg each of Carbosieve X and Carbosieve B; Supelco, Bellefonte, PA, USA). Isoprene from emitting lines was quantified following the protocol for volatile thermal desorption gas chromatography‐mass spectrometry (GC‐MS) (after Dani *et al*., 2017). Briefly, an Agilent 5975 gas chromatograph‐mass spectrometer (Agilent Technologies, Santa Clara, CA, USA) system was fitted with an HP‐InnoWax (50 m length, 0.2 mm inner diameter, 0.4 µm film) column. Thermal desorption was executed by a Twister® multipurpose autosampler and TD unit (Gerstel Technologies, Mülheim, Germany) fitted with an e‐Trap cryofocussing system (Chromtech‐Analytical Instruments, Bad Camberg, Germany). The GC separation programme was 40°C for 1 min, reaching 110°C at 5°C min^−1^, held for 10 min, and then increased to 260°C at 30°C min^−1^ and held for 2 min. Isoprene standards were prepared in 2 l Tedlar bags (Sigma‐Aldrich, St Louis, MO, USA) containing nitrogen, and analysed as earlier. Samples collected from the cuvette headspace containing control lines were treated as zero‐isoprene controls.

### Extraction and quantification of cytokinins

Cytokinins were extracted from fully expanded *Arabidopsis* leaves (position 7–10) before flowering under low‐light (at 36 and 48 DAG) and high‐light treatments (at 28 and 36 DAG). For *Arabidopsis*, leaves from at least four individuals were pooled to get one biological replicate with sufficient leaf mass and four such biological replicates were analysed. This meant ≥ 10 individuals per line for each treatment group were sampled. CKs were also extracted from fully mature healthy leaves (45–60 DAE) in all poplars. Cut portions of two to three poplar leaves (of similar age) from one individual was pooled to get one true biological replicate and four such biological replicates were analysed. CKs were extracted in acidified aqueous methanol (MeOH), purified by two solid‐phase extraction (SPE) steps and subsequently measured with a high‐performance liquid chromatography coupled to tandem mass spectrometry (LC‐MS/MS; Applied Biosystems, Waltham, MA, USA, as in Schäfer *et al*., 2014; also see Dobrev & Kaminek, 2002). Then, 30–400 mg ground plant tissue was extracted twice with 800 µl methanol : water : formic acid (15 : 4 : 1) at −20°C. Deuterated internal CK standards in the form of 0.2 ng [^2^H_6_] iPR, 0.2 ng [^2^H_5_] tZR, and 1 ng [^2^H_5_] tZ were supplemented in the first extraction step (standards from OlChemIm s.r.o., Olomouc, Czech Republic). The expanded hormone abbreviations for internal standards are: iPR, isopentenyladenine riboside; iP, isopentenyladenine; and tZR, trans‐zeatin riboside. Also see the caption under Fig. 2; Supporting Information Table S1 for additional details. Extraction and SPE were performed in 96 Well BioTubes (ArcticWhite LLC, Bethlehem, PA, USA) and 96‐Well Deep Well Plates (Thermo Scientific, Waltham, MA, USA). The first SPE step was performed on a Multi 96 HR‐X column (Macherey‐Nagel, www.mn‐net.com/us/chromatography/) conditioned with extraction buffer. The flow through was collected and the MeOH was evaporated at 42°C under constant nitrogen flow. Then, 850 µl of 1 M HCOOH was added to the samples and loaded on a Multi 96 HR‐XC column (Macherey‐Nagel) pre‐conditioned with 1 M HCOOH. Sequentially 1 ml each of 1 M HCOOH, MeOH, 0.35 M ammonium hydroxide (NH_4_OH) were added and eluted. Finally, CKs were eluted with 1 ml 0.35 M NH_4_OH in 60% MeOH. The second SPE was performed using a Chromabond Multi 96 vacuum chamber. After evaporation, samples were reconstituted in 50 µl 0.1% acetic acid, CKs were chromatographically separated on a Zorbax Eclipse XDB‐C18 column (50 mm × 4.6 mm, 1.8 µm) at 25°C fitted to an Agilent 1200 HPLC system (Agilent Technologies). Solvent A (water, 0.05% HCOOH) and solvent B (acetonitrile) mixture was supplied at 1.1 ml min^−1^ (0–0.5 min, 95% A; 0.5–5 min, 5–31.5% B in A; 5–6.5 min, 100% B; and 6.5–9 min 95% A). The LC was coupled to an API 6500 tandem mass spectrometer (AB Sciex, Darmstadt, Germany) equipped with a Turbospray ion source and quadrupole mass analyser. Data was acquired and processed using Analyst 1.6.3 software (AB Sciex). The MS was in positive ionization mode (MRM modus) to monitor analyte parent to product ion conversion (Table S1). We did not have internal standards for and hence could not quantify the immediate CK‐nucleotide precursors of active iP and tZ. Hence, those are not reported. CK‐riboside levels in *Arabidopsis* leaves sampled just before flowering are given in Table S2.

### RNA extraction, library preparation, RNA‐sequencing and differential gene expression analysis

Fully‐expanded *Arabidopsis* leaves in position 8, 9 and 10 (from the base) were sampled from eight individuals per each line before flowering, i.e. 36–40 DAG under low‐light treatment, and 28–36 DAG under high‐light treatment. Summer leaves from poplars were sampled from four individuals (per line) at 60 and 90 DAE. Leaves were frozen in liquid nitrogen and stored at −80°C. *Arabidopsis* leaves were pooled (2–3 individuals forming one biological replicate) to get sufficient starting material for RNA extraction. Leaves from two *Arabidopsis* lines (NE EV‐B3 control and IE ISPS‐C4) and two poplar lines (emitting WT control and isoprene‐suppressed RA1) were used for RNA‐sequencing. Sampled leaves were ground in liquid nitrogen and total RNA was extracted from *c*. 100 mg of ground leaf material (three biological replicates per genotype, per leaf life‐stage) using the mirPremier Isolation Kit (Sigma‐Aldrich) following the manufacturer’s instructions. Ribosomal RNA depleted strand‐specific RNA libraries were generated (in triplicate) using the Illumina TruSeq Stranded Total RNA library preparation kit with Ribo‐Zero Plant (Illumina Inc., San Diego, CA, USA). Libraries were quantified using Agilent 2100 Bioanalyser RNA assay (Agilent Technologies), pooled in equimolar amount and sequenced on a single lane on the Illumina NovaSeq 6000 sequencer to get 150 bp paired‐end reads (2 × 15 million total reads). Images from the instruments were processed following the manufacturer’s software pipeline to generate Fastq sequence files. Adaptor sequences and low quality 3′ ends were removed from short reads using, respectively, Cutadapt (Martin, 2011) and Erne‐Filter (Del Fabbro *et al*., 2013). After trimming, only pairs with both reads longer than 50 bp were retained. Trimmed reads were aligned against the *Populus tremula* v.2.2 reference genome (ftp://plantgenie.org/Data/PopGenIE/Populus_tremula/v2.2) and the *Arabidopsis thaliana* (TAIR10) reference genome (http://www.Arabidopsis.org) using Star v.2.7.2b (Dobin *et al*., 2013) with default parameters. The htseq‐count python utility (Anders *et al*., 2015) was used to calculate gene‐based read count values considering only uniquely mapping reads. The HTSeq count data were used as the input to measure differential gene expression using the Bioconductor package DESeq2 v.1.14.1 (Love *et al*., 2014) implemented in R. The raw counts of each gene were normalized to adjust for different sequencing depths across samples.

### Statistical analysis

Normality of observed values (within lines) was tested using the Kolmogorov–Smirnov test. Differences in means among lines for net photosynthesis (CO_2_ assimilation rate), electron transport rate, stomatal conductance, above ground biomass, and inflorescence weight (only *Arabidopsis*) were tested by one‐way analysis of variance (ANOVA) followed by a *post hoc* Tukey’s multiple comparison test (*n ≥* 10 biological replicates per line, *α* = 0.05). Differences in poplar photosynthesis and chloroplast energy status were tested using data collected from ≥ 5 leaves per individual at each sampled leaf age (*n ≥* 6 biological replicates per line). Differences in isoprene emission rate and CK abundance was verified by either Kruskal–Wallis *H* test for comparing medians (*n ≥* 4; *α* = 0.05) or by Games–Howell test for comparing means when variances were unequal (*n ≥* 4; *α* = 0.05). Leaf sampling details are given under their respective sub‐headings under methods. Differentially expressed genes (from deseq) were identified using pairwise comparisons at an adjusted *P*‐value (false discovery rate, FDR) threshold of ≤ 0.01 for determining significance. Transcripts with log_2_(fold change) > 1.5 and < −1.5 at *P*_adj_ < 0.001 were considered biologically significant, truly sensitive to presence or absence of isoprene, and shortlisted for functional interpretation. The fold change heatmap was generated using the open‐source Morpheus (https://software.broadinstitute.org/morpheus) matrix visualization application. All other statistical tests were carried out using Minitab 18.1 statistical package (Minitab Inc., State College, PA, USA).

## Results and Discussion

### Isoprene enhances leaf cytokinin metabolism, accelerates plant growth rate, strengthens apical dominance, and induces early flowering, early leaf and plant senescence

**Fig. 1:**
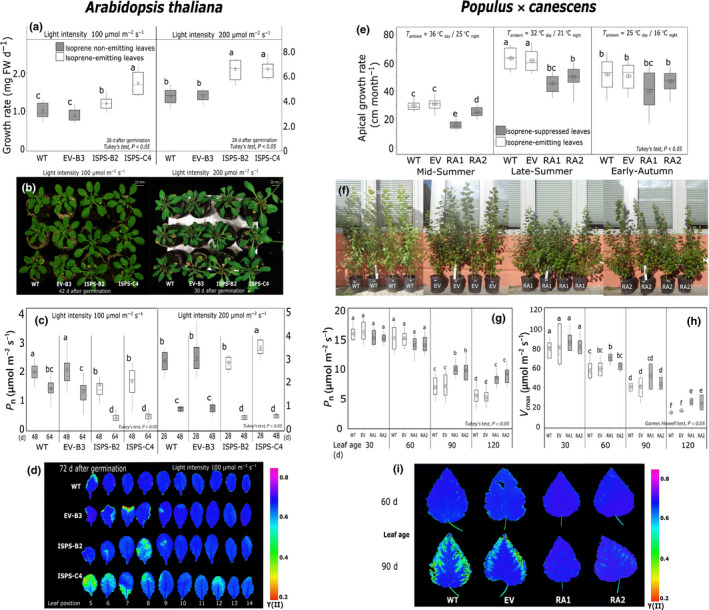
Growth rate and leaf senescence course in (a–d) isoprene‐emitting (white columns) and nonemitting (grey‐columns) *Arabidopsis* and (e–i) isoprene‐emitting (white columns) and isoprene‐suppressed (grey‐columns) poplar. For *Arabidopsis*, the responses of plants grown under low‐light (100 μmol photons m^−2^ s^−1^) and under high‐light (200 μmol photons m^−2^ s^−1^) are compared. (a) Growth rate for the first 28 d after germination (DAG), (b) photographs showing aboveground plant phenotype, (c) plant age‐specific net photosynthesis (*P*_n_) in fully mature and senescing *Arabidopsis* leaves, (d) representative chlorophyll fluorescence images showing the quantum yield of photosystem II (Y(II)) and early senescence in isoprene‐emitting leaves of *Arabidopsis* (72 DAG). For poplars, (e) apical growth rate (rate of change in the main stem height) in mid‐summer (July), late‐summer (August–September), and early‐autumn (October), (f) plant phenotype in 10‐month‐old saplings. (g) Leaf age‐specific *P*_n_ and (h) maximum carboxylation rate by Rubisco (*V*_cmax_) for leaves at 30, 60, 90 and 120 d after emergence (DAE), (i) chlorophyll fluorescence‐based estimation of maximum photochemical Y(II) for summer‐leaves of age 60 and 90 DAE. Six to 10 individual plants per line (true biological replicates) were sampled for both *Arabidopsis* and poplar. See methods for details of technical replicates. The box for each plant type in each plot includes the mean (circle), median (horizontal line), and the box spans lower and upper quartiles. The whiskers span the full data range. Tukey’s test, *α* = 0.05. Means that are significantly different do not share alphabetical letter codes. WT, wild‐type (white bars); EV‐B3 (*Arabidopsis*) and EV (poplar), empty vector control (grey bars); ISPS‐B2 and ISPS‐C4, transgenic isoprene‐emitting *Arabidopsis* lines (white bars); RA1 and RA2, transgenic isoprene‐suppressed (nearly nonemitting) poplar lines (grey bars).

**Fig. 2:**
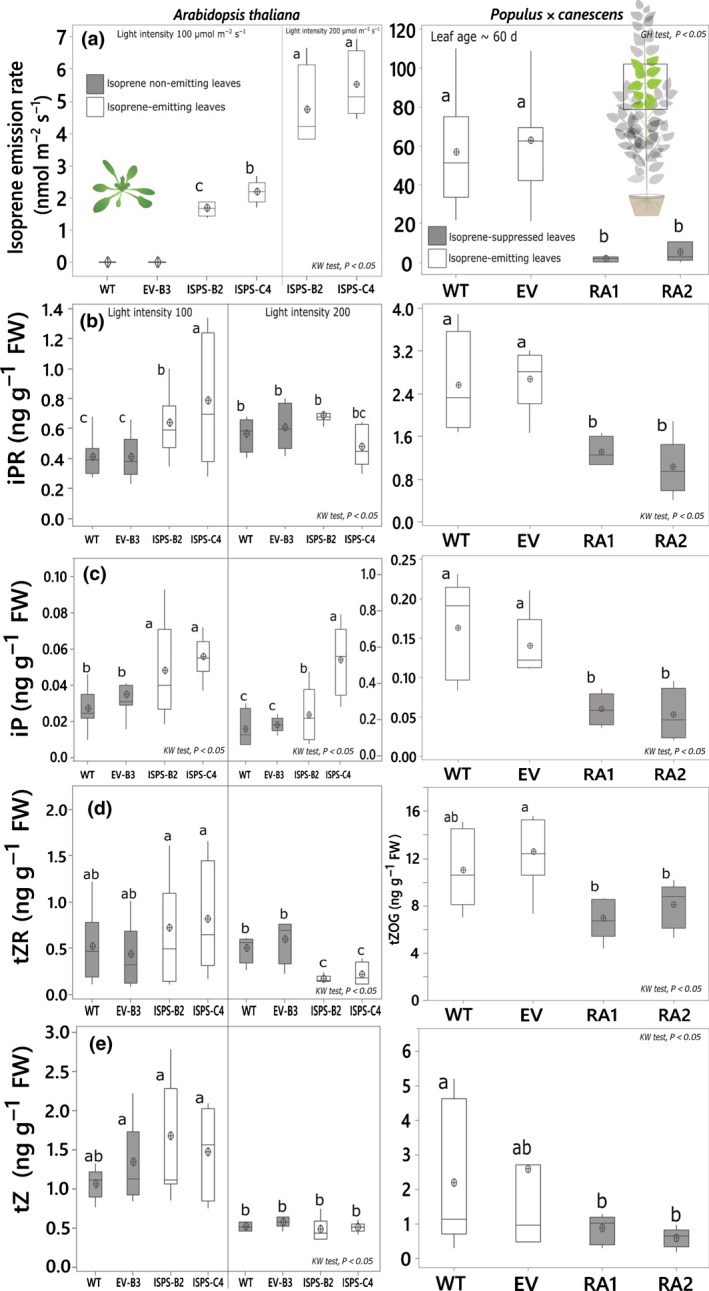
Isoprene emission rate and cytokinin (CK) concentration in *Arabidopsis* (left panels) and poplar leaves (right panels). Measurements from both low‐light (100 μmol photons m^−2^ s^−1^) and high‐light (200 μmol photons m^−2^ s^−1^) treatments are shown for *Arabidopsis*. (a) Isoprene emission rate, (b) isopentenyladenine riboside (iPR), (c) isopentenyladenine (iP), (d) trans‐zeatin riboside (tZR) for *Arabidopsis* and trans‐zeatin‐O‐glucoside (tZOG) for poplars, (e) trans‐zeatin (tZ). Leaves from at least four individual plants per line (biological replicates) were pooled to get one true replicate and four such pooled replicates per line were analysed in *Arabidopsis*, which meant that > 10 individuals were sampled per line. *Arabidopsis* leaves sampled were 36–48 d old (low‐light), 28–36 d old (high‐light), all before flowering, and were maintained at 20°C during sampling. In poplars, cut parts of two to three leaves per individual (per line) were pooled to get one biological replicate and four such true biological replicates (individuals) were sampled. Poplar leaves were 45–60 d old, and were maintained at a light intensity of 1500 μmol photons m^−2^ s^−1^ before and during sampling. The horizonal line within the box corresponds to the median and the box marks the lower and upper quartiles. Circles with + symbol mark the mean. Statistical significance of differences in isoprene emission rate were ascertained by one‐way ANOVA followed by a *post hoc* Tukey’s test. For CKs, both Kruskal–Wallis (KW) *H* test for medians and ANOVA followed by Games–Howell (GH) test for equality of means were applied (all *α* = 0.05). Means that are significantly different do not share alphabetical letter codes. WT, wild‐type (white bars); EV‐B3 (*Arabidopsis*) and EV (poplar), empty vector control (grey bars); ISPS‐B2 and ISPS‐C4, transgenic isoprene‐emitting *Arabidopsis* lines (white bars); RA1 and RA2, transgenic isoprene‐suppressed (nearly nonemitting) poplar lines (grey bars).

**Fig. 3:**
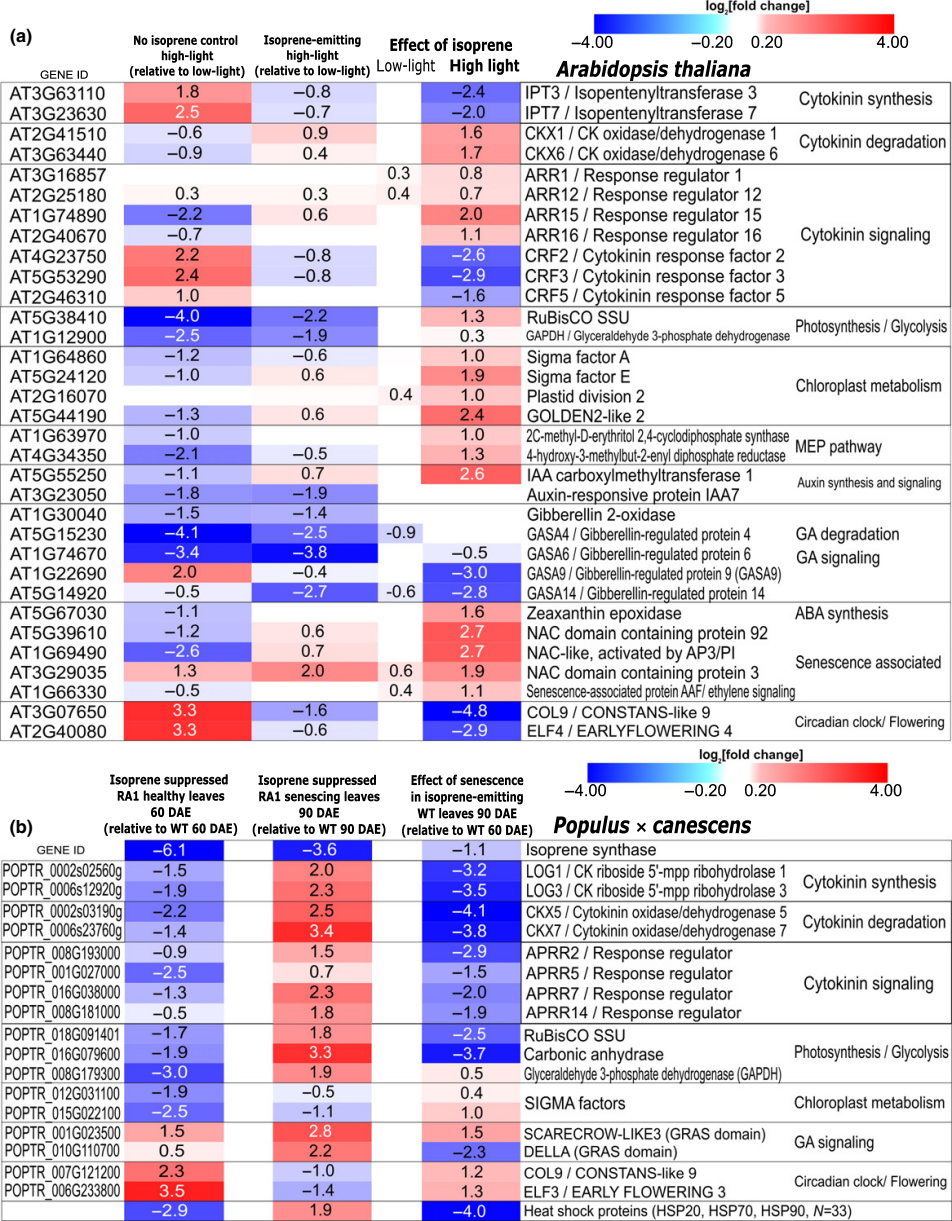
Impact of isoprene emission in *Arabidopsis* (a), and isoprene suppression in poplar (b) on the differential expression of genes involved in cytokinin synthesis, cytokinin degradation, cytokinin signalling, photosynthesis, chloroplast metabolism, noncytokinin hormone signalling, senescence associated transcription factors and flowering. Most significant log_2_(fold change) in transcript abundance during pairwise comparisons are shown. Darker the blue, more depleted are the transcripts and similarly brighter the red, more enriched are the transcripts. Wherever log_2_(fold change) is > +1.5 and < −1.5, the corresponding *P_adj_* is < 0.001. Wherever the fold change is less prominent but significant, the corresponding *P*_adj_ is often < 0.05. Nonsignificant changes are represented by white blanks without values. In panel (a), the change in transcript abundance is due to the presence of isoprene in isoprene‐emitting (IE) *Arabidopsis* leaves relative to nonemitting (NE) control except in column 1 where the effect of HL in NE leaves is indicated. The gene IDs correspond to the latest annotation of *Arabidopsis thaliana* genome (TAIR10). In panel (b), the change in transcript abundance is shown for healthy (60 d after leaf emergence) and senescing (90 d after emergence, DAE) leaves of isoprene‐suppressed (nearly NE) RA1 relative to IE control (WT). Darker blue represents depletion and brighter red represents enrichment in NE leaves at 60 and 90 DAE. In the third column of panel (b) comparison between senescing IE and healthy IE leaves shows changes in transcript abundance during natural senescence in IE control leaves. The gene IDs and chromosomal loci correspond to the latest annotation of the whole genome of *Populus trichocarpa* at the National Centre for Biotechnology Information (NCBI).

Isoprene‐emitting lines of *Arabidopsis* and poplar showed faster growth (Fig. 1a,b,e,f), early leaf senescence (monitored by early photosynthesis decline, Fig. 1c,d,g–i), and aboveground plant phenotype that was distinct from NE *Arabidopsis* and poplar lines (Fig. 1b,f). Significantly higher levels of iPR (Fig. 2b) and its free base derivative iP (Fig. 2c) in the IE leaves of both *Arabidopsis* and poplar with respect to NE leaves show that isoprene has a direct positive impact on the plastid‐localized synthesis of isoprenoid‐type CKs via the MEP pathway. Senescence associated declines in *P*_n_, *V*_cmax_, maximum photochemical yield of photosystem II (Y(II)), and instantaneous photosynthetic electron transport rate (ETR) occurred sooner and were steeper in IE than in NE *Arabidopsis* and poplar (Figs 1c,d,g, S1a). However, it is surprising that high CK abundance which is normally expected to delay senescence (Gan & Amasino, 1995; Werner *et al*., 2001), led to early leaf senescence in IE plants. While significant overexpression of genes coding for two‐component signal transduction/response regulators involved in CK‐signaling in IE leaves suggests greater CK activity, we also observed significant upregulation of cytokinin oxidase/deoxygenase in IE *Arabidopsis* (*AtCKX1* and *AtCKX6*, Fig. 3a) and poplar (*PtCKX5* and *PtCKX7*; Fig. 3b), indicating greater degradation of CKs. Leaf age‐specific changes in chloroplast energy status were altered during seasonal senescence in NE poplar leaves (Fig. S1b–d; not measured in *Arabidopsis*). SIGMA factors (facilitators of chloroplast RNA‐polymerase) and other transcription factors involved in chloroplast replication were enriched in IE *Arabidopsis* (Fig. 3a) and consistent with this, SIGMA factors and transcripts representing core‐photosynthetic genes were depleted in NE poplar leaves (Fig. 3b), all indicating faster chloroplast metabolic rate in presence of isoprene. We propose that faster cell metabolism and early senescence in IE leaves are driven by increased activity and recycling of CKs.

Isoprene‐emitting (IE) poplars showed a distinct within‐plant distribution of biomass due to their apically dominant phenotype (Fig. 1e,f; Table S3). Indeed, the *LOG* genes *PtLOG1* and *PtLOG3* (*LONELEY GUY* family), coding for cytokinin riboside 5′‐monophosphate phosphoribohydrolase involved in the activation of CKs and regulation of shoot apical meristematic growth (Kurakawa *et al*., 2007), were significantly overexpressed in the healthy mature leaves of apically dominant IE poplars (Fig. 3b). Weaker apical dominance and longer sylleptic branches in NE poplars (Fig. 1f) is potentially also linked to subdued activity of leaf cytokinine oxidases as indicated by depletion of *PtCKX* transcripts when isoprene is suppressed or absent (Fig. 3b). This is plausible since overexpression of *CKX* genes can increase CKX enzyme activity (Li *et al.*, 2019) and suppression of *CKX* genes can induce branching in different plant parts (Zhang *et al.*, 2021). IE leaves were richer than NE leaves in tZR (*Arabidopsis*), tZOG (trans‐zeatin O glucoside; poplar, Fig. 2d), and even cytosolic transfer RNA‐derived cZR (cis‐zeatin riboside; *Arabidopsis*, Table S2; also see Notes S1). Enlargement and thinning of IE leaves in *Arabidopsis* (Fig. S2d), and less consistently also in IE poplar, were likely triggered by altered zeatin ratios, which can affect leaf size (Osugi *et al*., 2017). It remains to be verified if leaf CK enrichment in IE leaves also affected the ratio between CK and auxins, the latter being a key regulator of apical dominance. Since iPR is transported in the phloem from leaves to all other meristems (Hirose *et al*., 2007), excess foliar iPR in IE leaves may cause faster tissue differentiation and expansion in all the organs of IE plants.

Isoprene‐emitting *Arabidopsis* flowered significantly earlier compared to NE control lines under both low‐light and high‐light (Figs 2c, S2e). Since high exogenous or endogenous CKs promote flowering and CK depletion delays flowering in annuals (Chaudhury *et al*., 1993; Corbesier *et al*., 2003), CK enrichment and heightened degradation of CKs in IE *Arabidopsis* leaves likely contributed to early‐flowering. Isoprene had either negative or no influence on genes involved in gibberellin (GA) signalling (Fig. 3a). Thus, CKs and not GAs likely induce early flowering in IE *Arabidopsis*, and perhaps also in plants fumigated with isoprene (Terry *et al*., 1995). *COL9* (*CONSTANS‐LIKE 9*) and *ELF4* (*ETS‐related transcription factor Elf‐4*) are negative regulators of flowering time (Doyle *et al*., 2002; Cheng & Wang, 2005; Kim *et al*., 2005), and their significant downregulation in IE *Arabidopsis* under high‐light may also explain longer hypocotyls (Zuo *et al*., 2019) and early flowering in IE *Arabidopsis*. The poplar *COL9* and *ELF3* transcripts were significantly enriched in NE leaves (Fig. 3b), which was consistent with their suppression in IE *Arabidopsis* leaves (Fig. 3a). Hence, isoprene and CK‐mediated changes in the circadian and photoperiodic signalling pathways likely contributed to early flowering and early senescence in IE plants. Given that isoprene interacts with stress‐responsive pathways (Zuo *et al*., 2019; Frank *et al*., 2021), early‐flowering in IE *Arabidopsis* could be an upshot of upregulated stress signalling.

### Isoprene associated cytokinin enrichment induces stress and defense response genes, with limited benefits under stress‐free conditions

The impact of isoprene on stress response, expression of genes involved in stress mitigation and less clearly also on growth are acknowledged in different experimental contexts (Behnke *et al*., 2010; Zuo *et al*., 2019; Monson *et al*., 2020; Xu *et al*., 2020; Frank *et al*., 2021). However, it is unlikely that isoprene played either thermo or photoprotective role in *Arabidopsis* grown at 18 ± 2°C under low‐light intensity (100 μmol photons m^−2^ s^−1^) since the photochemical parameters ETR and Y(II) of IE and NE *Arabidopsis* were similar (Fig. S3). Photosynthetic capacities of young and mature NE and IE poplar leaves were comparable even at relatively hot ambient temperatures > 35°C (Fig. 1g). Thus, the difference in the senescence course of IE and NE leaves was not caused by extrinsic stresses, where isoprene is known to be beneficial (Vickers *et al*., 2011; Way *et al*., 2011). It was not tested if changes in foliar hormonal balance due to isoprene suppression caused any ‘intrinsic stress’ that involved metabolically damaging changes in free radicle and redox status. Any intrinsic stress in NE leaves could have led to them senescing sooner than IE leaves, which was not the case.

**Fig. 4:**
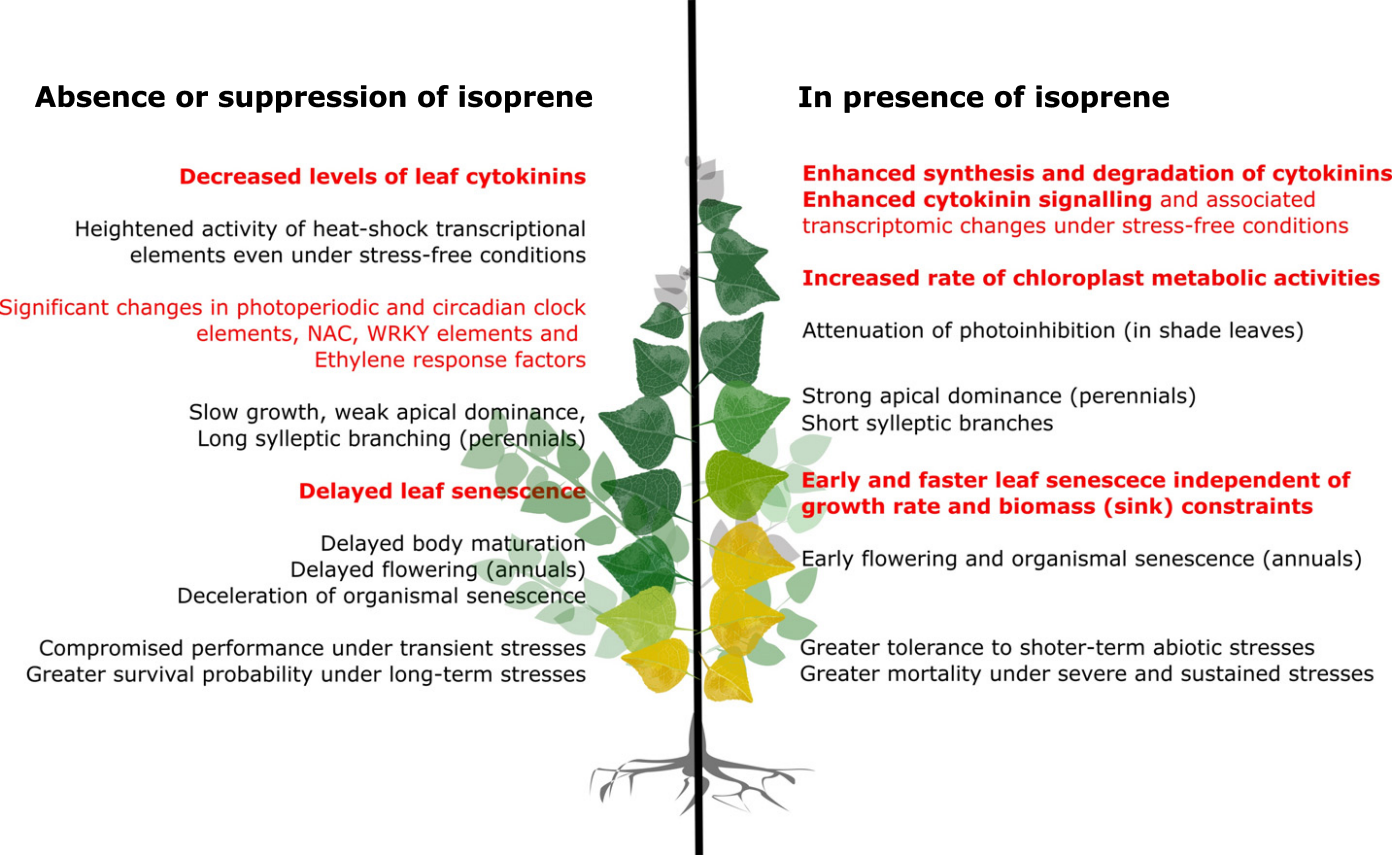
Illustration of metabolic and evolutionary consequences of isoprene emission capacity in plants. The statements in red are the most significant novel insights obtained from the current study, and the remaining facts (some but not all verified by other published studies) are also summarized.

In *Arabidopsis*, thinning of IE leaves under low‐light may have led to lower *P*_n_, but not when light‐intensity was increased (200 μmol photons m^−2^ s^−1^) while keeping all other conditions identical. However, one of the IE lines (ISPS‐C4) showed the highest isoprene emission rate and *P*_n_ under high‐light (Figs 1c, 2a) where expression of photosystem I and photosystem II light harvesting complexes was less suppressed relative to NE leaves (Fig. S4), indicating that isoprene favours photosynthetic stability when emission occurs at a reasonably high level even in plants acclimated to nonstressful but higher light intensity (Fig. 2a). A sweeping downregulation of heat shock proteins (HSPs, Fig. 3b), differential expression of NAC and WRKY transcription factors, and ethylene response factors (ERFs) involved in abiotic stress responses (Figs S5–S7) in unstressed NE poplar leaves broadly agreed with previous studies showing interactions between isoprene and stress‐response pathways (Behnke *et al*., 2010; Zuo *et al*., 2019; Xu *et al*., 2020). Noting that CKs themselves can impart photoprotective benefits, and since stress‐induced leaf senescence involves CK depletion and senescence is delayed by CK enrichment (Rivero *et al*., 2007; Cortleven & Schmülling, 2015), we attribute known tolerance for transient heat and high‐light stresses in IE leaves also to localized enhancement of foliar CK synthesis and activity. The concentration of active CK‐free base (iP) increased by 10 times in high‐light in all *Arabidopsis* lines (Fig. 2c), indicating stimulation of CK‐riboside to active CK‐freebase conversion by light, which was most pronounced and significant in IE leaves (Fig. 2c). Such high levels of iP (and no change in iPR; Fig. 2b) may have caused downregulation of leaf *AtIPT* genes (CK synthesis) in IE leaves under high‐light (Fig. 3a). However, as in low‐light, transcripts of *AtCKX* genes (CK degradation) were enriched in early‐senescing IE leaves under high‐light (Fig. 3a). Besides, sustained CK activity under prolonged stress can be detrimental (Nishiyama *et al*., 2011), and this, among other costs associated with fast‐growth, can make IE plants more susceptible to premature mortality under severe and prolong stresses (Fig. 4).

### Early leaf senescence in isoprene‐emitting poplars is an innate behaviour not driven by faster growth rate

Plant growth and reproductive strategies, i.e. growing fast or slow and flowering early or late, influence the timing and pace of leaf senescence at least in annuals, and the same was evident in IE *Arabidopsis*. Accelerated development and early flowering in IE *Arabidopsis* was associated with lighter aboveground vegetative body mass and early body (leaf) mass loss during senescence (Fig. S1d). It is difficult to verify the same in long‐lived trees. However, body size maturation and attainment of reproductive maturity may determine when and how whole‐plant senescence proceeds even in perennials (Dani & Kodandaramaiah, 2019). Although the total biomass of IE and NE poplars was comparable in our study (also in Way *et al*., 2011; Behnke *et al*., 2012; Monson *et al*., 2020), the early‐rising and apically dominant (light‐competitive) IE trees were expected to attain adulthood sooner and to undergo actuarial and demographic senescence earlier than closely related NE species. However, the role of fast or slow growth strategy on the timing and pace of autumn leaf senescence has not been explicitly tested in poplars. Seasonal leaf senescence, despite being a strongly heritable character preserved over generations in deciduous perennials, is sensitive to year‐on‐year variation in seasonal growth and environmental stress factors (Panchen *et al*., 2015; Zohner & Renner, 2017; Michelson *et al*., 2018; Richards *et al*., 2020). Timing of bud‐burst and leaf flushing correlate with growth rate, growth duration and biomass accumulation in poplars (Yu *et al*., 2001; Müller *et al*., 2013). However, spring‐sprouting occurred at the same time in all four poplar lines, which ruled out spring‐phenology influence on senescence regimes of IE and NE poplars. This was not surprising given endogenous isoprene is absent when trees are completely defoliated in winter, and barring unknown epigenetic memory effects, isoprene has limited realtime influence on spring sprouting. The impact of growth rate on leaf senescence was less clear and varied within individual plants. Leaf lifespan was shorter when the length of sylleptic branches was longer in NE poplars (Fig. S8c), which suggested that modular nutrient remobilization and relative strength of local sinks (adjacent to senescing nodal leaves) were key factors that determined which leaf senesced first and at what rate (Table S3; Notes S2; also see Sprugel *et al*., 1991). We note that IE leaves may have abscised early by the virtue of early senescence and *P*_n_ could not be monitored at high frequency to examine if IE leaves senesced faster and not just sooner. Instead, we tested whether the observed early senescence (early decline in age‐specific *P*_n_; Fig. 1g) in IE leaves on the main stem occurred as a consequence of faster apical growth and metabolism (proxied by chloroplast status) of IE poplars relative to NE poplars.

We imposed ‘no new growth’ condition on both IE and NE poplars where apical and axillary sprouts were pruned once and repeatedly until the end of the experiment in December. Decapitation and ‘imposed no‐growth’ treatment extended leaf lifespan by more than a month in all poplars confirming the importance of the strength of growing sinks in pacing leaf senescence. However, in apically intact poplars, an early decline in *P*_n_ was observed even in late‐emerging apical IE autumn leaves relative to contemporary NE leaves, both presumably not supplying remobilized nutrients to any significant growing sink late in the season (Fig. S8a). Even under ‘no‐growth’ scenario, *P*_n_ declined significantly sooner in senescing IE leaves than in senescing NE poplar leaves (Fig. S8d), and the early‐senescing phenotype of IE leaves was preserved (Fig. S9). These observations show that early‐ or late‐senescing tendency of leaves is governed by isoprene‐induced reprogramming of biological processes that are innate and distinct from those affecting seasonal sink constraints and growth rates. We attribute early senescence of IE leaves primarily to enhanced CK metabolism and CK‐targeted transcriptional regulation in presence of isoprene in unstressed condition, and other isoprene‐associated changes that are detectable under stressful conditions. The question, what about leaf senescence in those deciduous and evergreen trees that emit tiny amounts of isoprene and deciduous trees that do not emit isoprene at all, does arise. These trends need to be further tested in other deciduous IE tree genera that are typically faster‐growing and more diverse than related NE genera (Dani *et al*., 2014a). At this stage, isoprene emission seems to act as a pacemaker of leaf senescence when it occurs at significantly high rates, and absolute rates of emission may be less important than emission rates relative to the rate of photosynthesis and other demands in chloroplasts. There are limitations to studying seasonal senescence in juvenile poplars, which have different body architecture and growth constraints compared to adult trees. Nonetheless, the leaf level metabolic changes due to isoprene are likely under identical genetic and physiological constraints in both juveniles and adult trees in most species (including poplars), except perhaps in cases where tree maturation also alters leaf structure. Tissue‐specific metabolic constraints are as important as temperature, photoperiod, nutrient availability and organismal age in determining the timing and pace of seasonal senescence and lifespan of plant organs, which may not always influence the pace of whole‐plant senescence.

Global warming and CO_2_ fertilization by the end of this century are expected to either delay autumn senescence due to extended periods of favourable temperatures or cause early autumn senescence due to quicker growth and water‐limited conditions (Richardson *et al*., 2010; Chen *et al*., 2020; Zani *et al*., 2020). More isoprene could be released if growing seasons get longer and/or warmer in the future (Peñuelas *et al*., 2009; Sharkey & Monson, 2014). Our result, that isoprene‐emission changes CK homeostasis and induces early leaf senescence irrespective of growth rates and sink constraints, becomes an important factor that influences the course of autumn leaf senescence and limits the release of carbon as isoprene from deciduous forests.

### Conclusion

Isoprene‐emitting plants are more tolerant to transient abiotic stresses than nonemitters, but an overarching explanation for the relevance of constitutively emitted isoprene in unstressed plants was missing. We show that isoprene emission from unstressed leaves significantly enhances leaf CK synthesis and induces a remarkably consistent pattern of CK‐associated transcriptional regulation in both herbaceous and tree model species. Early leaf senescence in presence of isoprene and associated changes in CK metabolism suggests that even a constitutive plant volatile like isoprene has a nontrivial stimulatory impact on development, reproduction, and senescence. These results unveil a hitherto unknown significance for constitutive isoprene emission that (1) makes emitting‐species more competitive by hastening their development, and more critically (2) curtails leaf and plant lifespan by accelerating ageing and senescence (Fig. 4). Faster development, early maturation, early senescence and shortening of generation time due to isoprene emission likely confers a major evolutionary advantage in emitting plants by not only imparting transient stress‐resilience (well recognized) but more fundamentally by facilitating stress‐escape (shorter life cycle), faster species diversification and potential colonization of new favourable habitats.

The consequences of endogenous isoprene on plant development and phenotype appears remarkably similar to more‐widely studied stimulation of plant growth by various exogenous microbial volatiles (Junker & Tholl, 2013; Sharifi & Ryu, 2018). A key distinction is that continuous endogenous emission (and not exogenous exposure) incurs metabolic costs to the emitter, which may directly affect organellar carbon and energy status and hormone synthesis (our study) and also hormone‐mediated signalling (various studies). We anticipate that other endogenous volatiles, which are so far purely implicated in stress mitigation and inter‐organismal communication, will have significant interactions with canonical hormone‐mediated developmental pathways in unstressed plants (Dani & Loreto, 2022). Future experiments involving mutants of CK synthesis and signalling will shed more light on specific mechanisms of interference from isoprene and other biogenic volatiles in CK‐mediated changes in leaf and plant metabolism.

## Author contributions

KGSD conceived the hypothesis, visualized, designed, led the study, and conducted the experiments with FL. TDS and J‐PS provided the transgenic *Arabidopsis* and poplar lines, respectively. S Pollastri helped raising the *Arabidopsis* seed stock, along with FL, helped with logistics. MR extracted and quantified cytokinins. S Pinosio assembled the RNA‐sequencing raw reads and the transcript abundance data. KGSD monitored leaves from emergence until senescence, quantified leaf photosynthesis, chloroplast energy status, quantified isoprene emission, leaf and plant phenotype, analysed hormonal and differential gene expression data. KGSD wrote and revised the manuscript with inputs from FL and other authors.

## Supporting information


**Fig. S1** Chloroplast energy status of poplar leaves during senescence.
**Fig. S2** Quantum yield of photosystem II in *Arabidopsis*.
**Fig. S3** Growth and flowering in isoprene‐emitting *Arabidopsis*.
**Fig. S4** Isoprene and the expression of photosystem genes in *Arabidopsis*.
**Fig. S5** Isoprene and the expression of poplar NAC transcription factors.
**Fig. S6** Isoprene and the expression of poplar WRKY transcription factors.
**Fig. S7** Isoprene and the expression of poplar ethylene response factors (ERFs).
**Fig. S8** Net photosynthesis (*P*_n_) in apically intact and ‘no growth’ poplars.
**Fig. S9** Leaf photographs showing early senescence in apically intact and ‘no‐growth’ poplars.
**Notes S1** Supplementary note associated with Table S2.
**Notes S2** Supplementary note associated with Table S3.
**Table S1** Parameters of LC‐MS/MS analysis of cytokinins.
**Table S2** Cytokinin supplementary information including cis‐zeatin riboside (cZR) for *Arabidopsis*.
**Table S3** Poplar aboveground phenomic data.
Please note: Wiley Blackwell are not responsible for the content or functionality of any Supporting Information supplied by the authors. Any queries (other than missing material) should be directed to the *New Phytologist* Central Office.

## Data Availability

All data are available in the main text or the supplementary materials. The RNA‐sequencing data that support the findings of this study are deposited to Sequence Read Archive of the National Centre for Biotechnology Information (NCBI) and the concerned accession numbers are PRJNA694035 and PRJNA690701 and the URLs are https://www.ncbi.nlm.nih.gov/Traces/study/?acc=PRJNA694035; https://www.ncbi.nlm.nih.gov/Traces/study/?acc=PRJNA690701.
