# Supplementary material for: Isoprene enhances leaf cytokinin metabolism and induces early senescence
Source: New Phytol. 2021 Dec 20;234(3):961–74. doi: 10.1111/nph.17833 (PMC9300082; doi:10.1111/nph.17833)
Supplement: Supplementary file 1 — Fig. S1 Chloroplast energy status of poplar leaves during senescence. Fig. S2 Quantum yield of photosystem II in Arabidopsis. Fig. S3 Growth and flowering in isoprene‐emitting Arabidopsis. Fig. S4 Isoprene and the expression of photosystem genes in Arabidopsis. Fig. S5 Isoprene and the expression of poplar NAC transcription factors. Fig. S6 Isoprene and the expression of poplar WRKY transcription factors. Fig. S7 Isoprene and the expression of poplar ethylene response factors (ERFs). Fig. S8 Net photosynthesis (P n) in apically intact and ‘no growth’ poplars. Fig. S9 Leaf photographs showing early senescence in apically intact and ‘no‐growth’ poplars. Notes S1 Supplementary note associated with Table S2. Notes S2 Supplementary note associated with Table S3. Table S1 Parameters of LC‐MS/MS analysis of cytokinins. Table S2 Cytokinin supplementary information including cis‐zeatin riboside (cZR) for Arabidopsis. Table S3 Poplar aboveground phenomic data. Please note: Wiley Blackwell are not responsible for the content or functionality of any Supporting Information supplied by the authors. Any queries (other than missing material) should be directed to the New Phytologist Central Office. [file NPH-234-961-s001.pdf]

New Phytologist Supporting Information

Article title: Isoprene enhances leaf cytokinin metabolism and induces early senescence

Authors: Kaidala Ganesha Srikanta Dani*, Susanna Pollastri, Sara Pinosio, Michael Reichelt, Thomas D Sharkey, Jörg-Peter Schnitzler, Francesco Loreto

article acceptance date: 22 October 2021

*Corresponding author. Email: srikantadani@yahoo.co.uk

The following Supporting Information is available for this article

Fig. S1 Leaf age-specific changes in chloroplast energy status of summer-emerging poplar leaves from the stage of leaf maturation to late-senescence

Fig. S2 Chlorophyll fluorescence imaging-based estimate of quantum yield of photosystem II in NE and IE *Arabidopsis* lines.

Fig. S3 Growth and flowering response of isoprene-emitting (IE) and non-emitting (NE) *Arabidopsis*.

Fig. S4 Impact of high-light (200 μmol m^{-2} s^{-1}) and isoprene-emission on the abundance of mRNA transcripts coding for thylakoid membrane-bound photosystems (PS I and PS II) in *Arabidopsis*

Fig. S5 Impact of isoprene-suppression on the abundance of mRNA transcripts coding for NAC transcription factors in poplars.

Fig. S6 Impact of isoprene-suppression in poplar leaves on WRKY transcription factors implicated in regulation of plant developmental and senescence pathways.

Fig. S7 Impact of isoprene-suppression in poplar leaves on Ethylene Response Factors (ERFs) implicated in regulation of plant stress response pathways

Fig. S8 Contrast between age-specific decline in net photosynthesis (P_n) in apically intact and "no growth" poplars

Fig. S9 Photographs showing conservation of early leaf senescence in isoprene-emitting (WT and EV) poplars in apically intact and "no-growth" condition

Table S1 Parameters of LC-MS/MS analysis of cytokinins in positive mode (see methods for details)

Table S2 Abundance of isopentenyladenine riboside (iPR) and cis zeatin-riboside (cZR) in *Arabidopsis* leaves sampled before flowering respectively for each line.

Table S3 Poplar above-ground phenomic data

Notes S1 Supplementary note associated with Table S2

Notes S2 Supplementary note associated with Table S3

Fig. S1

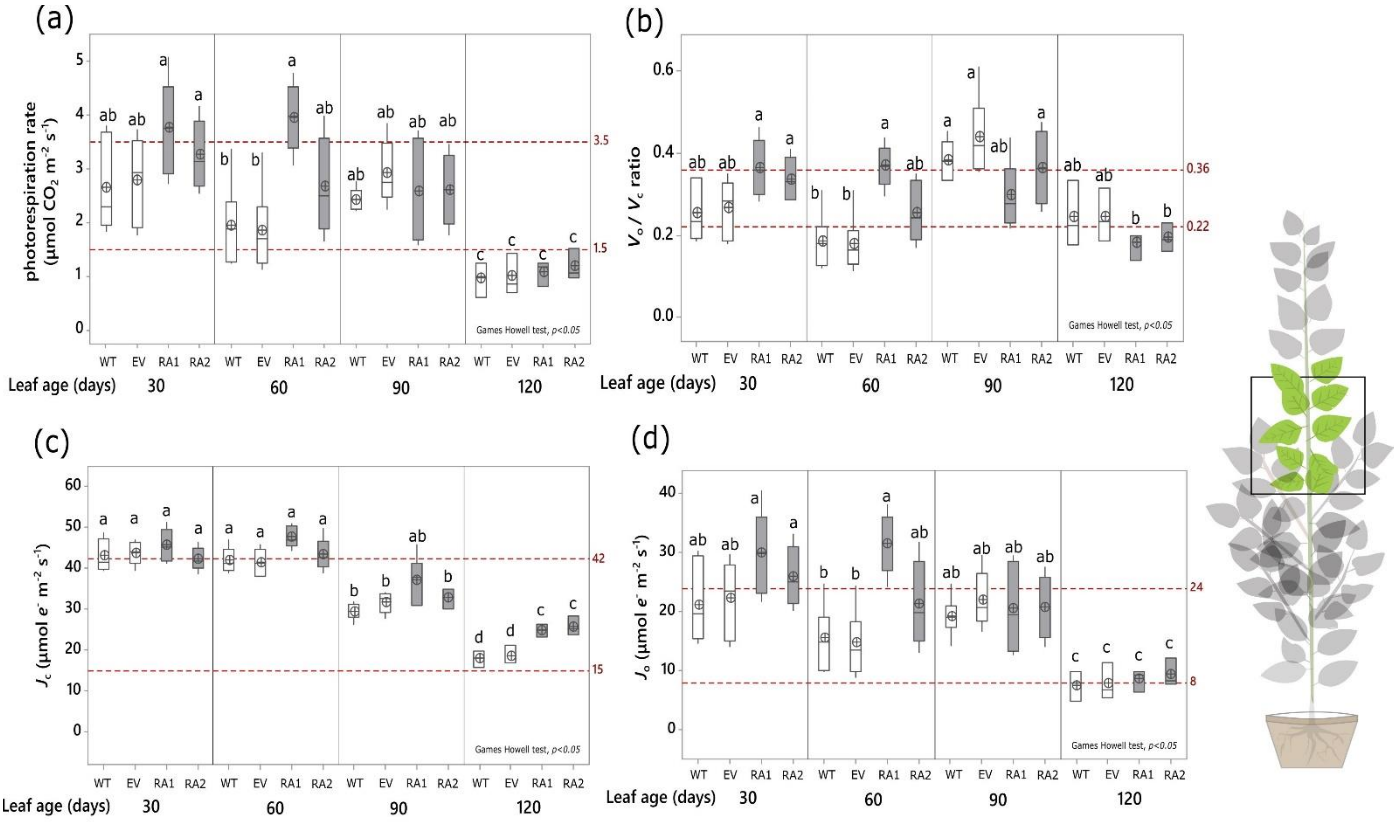

Fig. S1: Leaf age-specific changes in chloroplast energy status of summer-emerging poplar leaves from the stage of leaf maturation to late-senescence (a) Photorespiration rate, (b) Ratio between velocity of oxygenation and carboxylation (v_o/v_c), (c) Electron transport rate invested in photosynthetic carbon reduction (J_c), and (d) Electron transport rate available for photorespiration (J_o) measured in IE (white bars) and NE (grey bars) leaves of age 30, 60, 90 and 120 days. In total, six leaves of same age (one or two leaves each from four individual plants) per line were sampled at each stage, except at the final stage (at 120 days) when three individuals were sampled per line. The box for each line includes the mean and the box spans lower and upper quartiles. (Tukey's test, α=0.05). Means that are significantly different do not share alphabetical letter codes.

Fig. S2

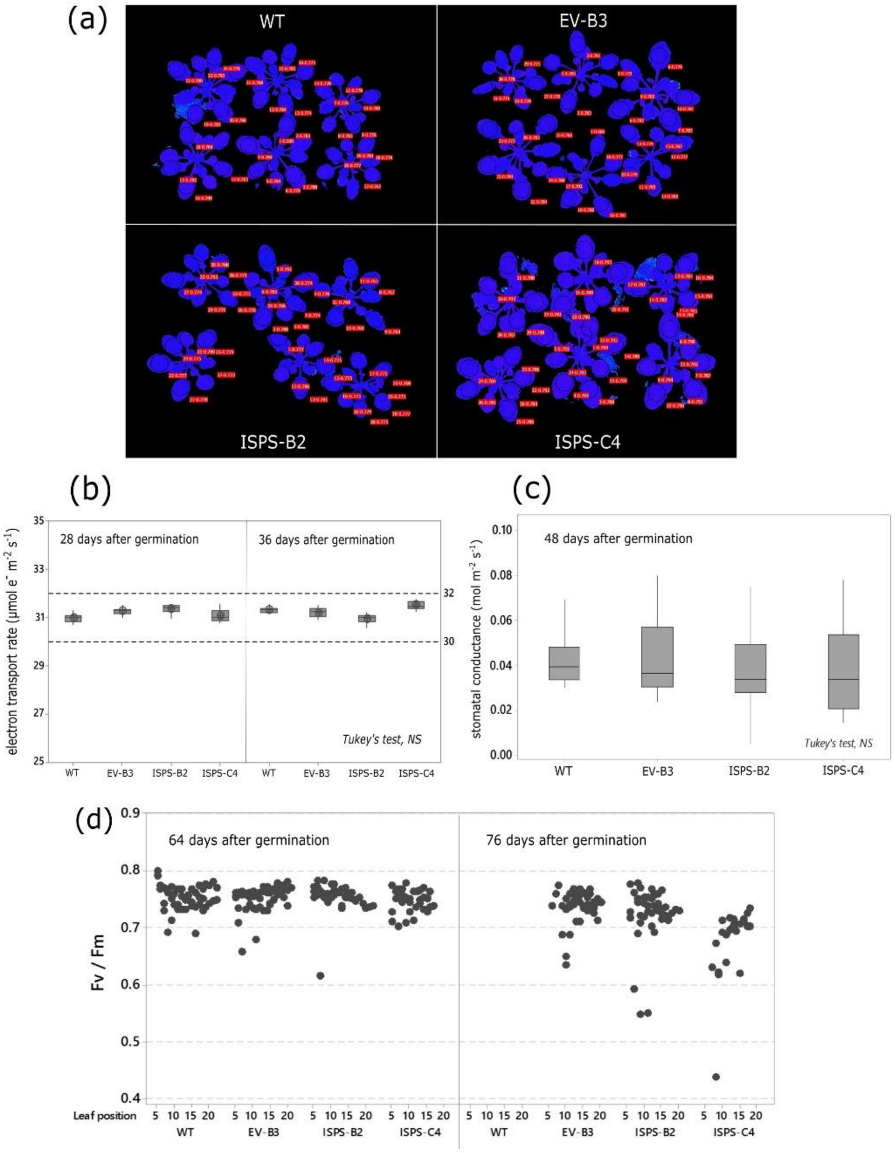

Fig. S2. (a) Chlorophyll fluorescence imaging-based estimate of quantum yield of photosystem II in NE (top panels) and IE (bottom panels) *Arabidopsis* lines. (b) Electron transport rate of IE and NE *Arabidopsis* lines 28 DAG (left) and 36 DAG (right). (c) Stomatal conductance of IE and NE *Arabidopsis* lines 48 DAG. (d) Maximum quantum yield of chlorophyll fluorescence (F_v/F_m) measured by leaf position in IE and NE *Arabidopsis* at 64 (left) and 76 DAG (right). The plants were acclimated to a light intensity of 100 μmol photons m^{-2} s^{-1}. For panels (b) and (c), boxes include the mean (circle), median (horizontal line), and span lower and upper quartiles. The whiskers span the full data range. N = 6 to 10. Tukey's test, $\alpha = 0.05$. NS = means are not significantly different.

Fig. S3

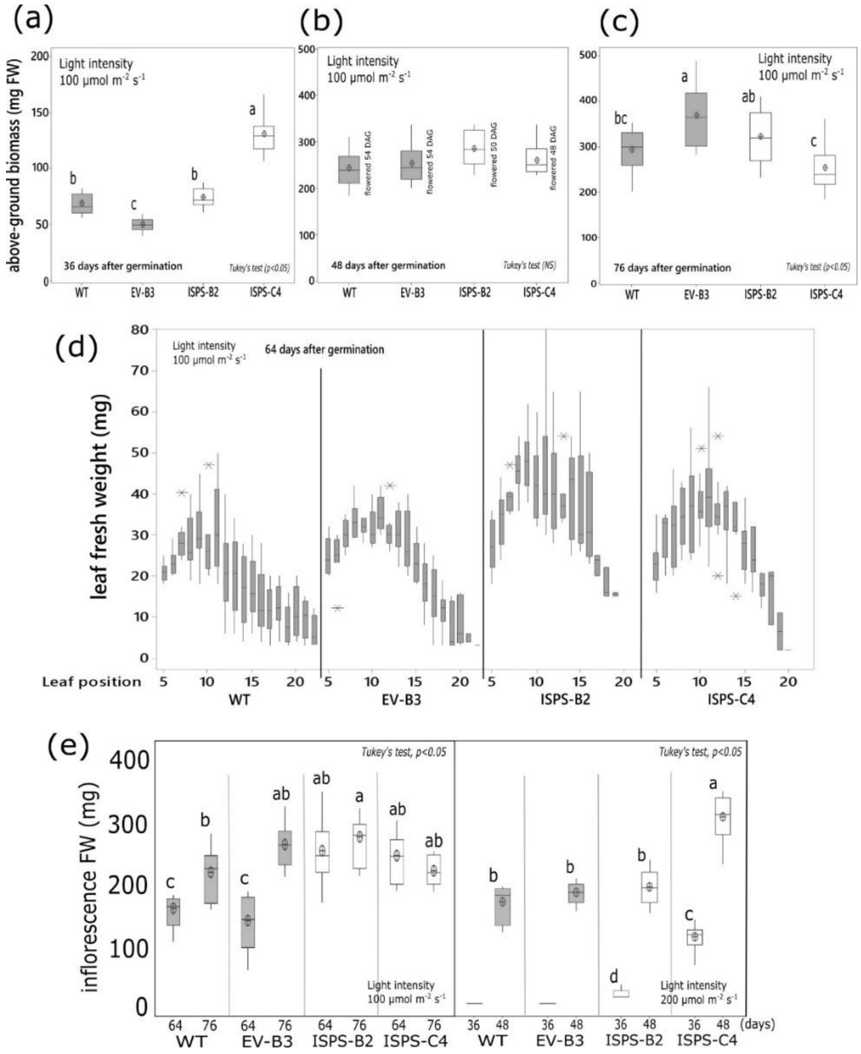

Fig. S3. Growth and flowering response of isoprene-emitting (IE) and non-emitting (NE) *Arabidopsis*. Above-ground biomass of IE lines ISPS-B2 and ISPS-C4 (white bars), and NE controls (grey bars) at (a) 36 DAG; (b) 48 DAG, and (c) 74 DAG. (d) Leaf-size (mass) distribution of IE and NE lines by leaf position at 64 DAG, and (e) Inflorescence growth in IE and NE lines under low-light (100 µmol photons m^{-2} s^{-1}, left panel) and high-light (200 µmol photons m^{-2} s^{-1}, right panel).

Fig. S4

Control (200) vs Control (100)	Isoprene (200) vs Isoprene (100)	Isoprene (100) vs Control (100)	Isoprene (200) vs Control (200)		GENE ID
-3.7	-2.5		1.1	photosystem II subunit T	AT3G21055
-2.5	-1.1		1.2	photosystem II subunit R	AT1G79040
-3.0	-1.8		1.1	photosystem II subunit QA	AT4G21280
-2.5	-1.4		1.2	photosystem II subunit Q-2	AT4G05180
-0.8	-0.6	0.5	1.1	photosystem II subunit P-2	AT2G30790
-2.8	-1.6		1.2	photosystem II subunit O-2	AT3G50820
-2.8	-1.6		1.4	photosystem II reaction center W	AT2G30570
-3.0	-1.5		1.2	photosystem II oxygen-evolving complex 1	AT5G66570
-3.3	-1.5		1.7	photosystem II light harvesting complex gene B1B2	AT2G34420
-5.6	-2.5	0.4	2.0	photosystem II light harvesting complex gene 2.3	AT3G27690
-4.2	-2.1	0.3	2.2	photosystem II light harvesting complex gene 2.2	AT2G05070
-4.7	-1.4	0.3	3.5	photosystem II light harvesting complex gene 2.1	AT2G05100
-3.6	-1.5		1.8	photosystem II light harvesting complex	AT3G08940
-2.6	-1.5		1.1	photosystem II BY	AT1G67740
-3.5	-1.5		1.8	photosystem II 5 light harvesting complex	AT4G10340
-3.4	-1.8		1.8	photosystem II 5 kD protein	AT1G51400
-3.0	-1.6		1.5	photosystem I subunit O	AT1G08380
-3.5	-1.9		1.6	photosystem I subunit K	AT1G30380
-3.1	-1.7		1.6	photosystem I subunit H2	AT1G52230
-3.4	-2.1		1.1	photosystem I subunit G	AT1G55670
-3.2	-1.7		1.6	photosystem I subunit F	AT1G31330
-3.2	-1.3		1.8	photosystem I subunit D-2	AT1G03130
-2.8	-1.3		1.5	photosystem I subunit D-1	AT4G02770
-3.6	-1.8		1.7	photosystem I reaction centre subunit IV / PsaE protein	AT4G28750
-3.3	-1.8		1.6	photosystem I reaction center subunit PSI-N, chloroplast	AT5G64040
-3.4	-1.5		1.7	photosystem I light harvesting complex gene 3	AT1G61520
-3.1	-1.5		1.5	photosystem I light harvesting complex gene 2	AT3G61470
-3.4	-1.9		1.4	photosystem I light harvesting complex gene 1	AT3G54890
-3.7	-1.4		2.1	light-harvesting chlorophyll-protein complex I subunit A4	AT3G47470
-5.1	-2.5		1.9	light-harvesting chlorophyll B-binding protein 3	AT5G54270
-6.4	-2.4		3.5	light harvesting complex- LHCIIb- chlorophyll A/B-binding protein 2	AT1G29920
-1.8	-0.8		1.0	high chlorophyll fluorescence phenotype 173	AT1G16720

log_2[Fold Change]: -6.00 — 0.00 — 3.00

Fig. S4: Impact of high-light (200 µmol m^{-2} s^{-1}) and isoprene-emission on the abundance of mRNA transcripts coding for thylakoid membrane-bound photosystems (PS I and PS II) in *Arabidopsis.* Isoprene-emission attenuates inhibition of photosystems (without exception) by high-light. Darker the blue, more depleted are the transcripts and similarly brighter the red, more enriched are the transcripts. Wherever log_2[fold change] is > +1.5 and <─1.5, the corresponding p_{adj} is <0.001. Wherever the fold change is less prominent but significant, the corresponding p_{adj} is often <0.05. Non-significant changes are represented by white blanks without values.

Fig. S5

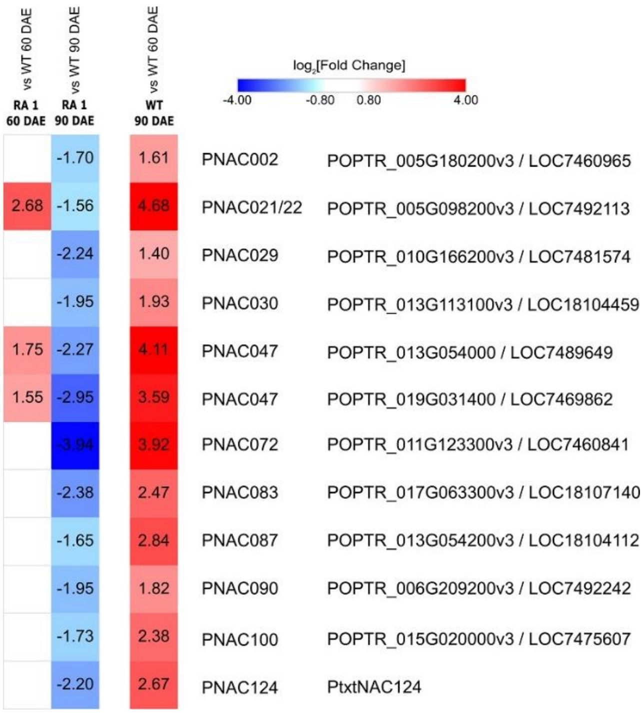

Fig S5: Impact of isoprene-suppression on the abundance of mRNA transcripts coding for NAC transcription factors in poplars. Column 1 shows that two NAC factors (PNAC 021/22 and PNAC 047) are significantly upregulated by isoprene-suppression in healthy RA1 leaves compared to healthy isoprene-emitting WT leaves (leaf age = 60 days after emergence). PNAC 021 has an *Arabidopsis* homolog named AtNAC014, which is a membrane-bound NAC factor, potentially involved in negative regulation of cell-division rate, and sensitive to cytokinin levels. Upregulation of PNAC 021 in isoprene-suppressed leaves is thus consistent with lower apical growth rate of NE poplars. Column 2 shows that the expression of all NAC factors is significantly lower in senescing RA1 leaves compared to senescing WT leaves (leaf age 90 days). Column 3 shows that all the NAC factors are significantly overexpressed in senescing WT leaves that emit less isoprene relative to healthy WT leaves. Darker the blue, more depleted are the transcripts and similarly brighter the red, more enriched are the transcripts. Wherever $\log_2$[fold change] is > +1.5 and <−1.5, the corresponding p_{adj} is <0.001. Wherever the fold change is less prominent but significant, the corresponding p_{adj} is often <0.05. Non-significant changes are represented by white blanks without values.

Fig. S6

log$_2$[Fold Change]: -4.00, -0.80, 0.80, 4.00

RA 1 60 DAE vs WT 60 DAE	RA 1 90 DAE vs WT 90 DAE	WT 90 DAE vs WT 60 DAE		Gene ID /Locus
1.45			WRKY transcription factor 7	POPTR_014G024200v3 / LOC18104849
	-2.06	4.43	WRKY transcription factor 27	POPTR_004G072000v3 / LOC7468350
1.54	1.62	2.81	WRKY transcription factor 31	POPTR_004G007500v3 / LOC7479058
1.95		-1.04	WRKY transcription factor 31	POPTR_014G155100v3 / LOC7462063
	1.88		WRKY transcription factor 40	POPTR_006G263600v3 / LOC7489150
	-1.52	2.68	WRKY transcription factor 41	POPTR_001G092900v3 / LOC7472714
-1.69	-3.03	5.57	WRKY transcription factor 43	POPTR_015G099200v3 / LOC7463139
	2.89	-1.77	WRKY transcription factor 46	POPTR_002G168700v3 / LOC7457389
-1.78	1.87		WRKY transcription factor 51	POPTR_007G079800v3 / LOC7457138
-1.63	2.42	-0.88	WRKY transcription factor 52	POPTR_005G085200v3 / LOC7486341
	1.95	-1.44	WRKY transcription factor 57	POPTR_010G160100v3 / LOC7492890
	3.07	-1.56	WRKY transcription factor 70	POPTR_006G109100v3 / LOC7484630
	-1.84	2.79	WRKY transcription factor 72	POPTR_015G064100v3 / LOC18105808
	-1.68	4.72	WRKY transcription factor 75	POPTR_T043800v3 / LOC18107219
-1.90	-2.46	0.92	WRKY transcription factor 75	POPTR_001G058800v3 / LOC18094503
	-1.68	2.22	WRKY TF unknown	POPTR_001G328000
2.15		2.12	WRKY TF unknown	POPTR_014G119800
	-2.37	3.15	WRKY TF unknown	POPTR_001G099001

Fig. S6: Impact of isoprene-suppression in poplar leaves on WRKY transcription factors implicated in regulation of plant developmental and senescence pathways. Column descriptions are as given in Fig. S5. Darker the blue, more depleted are the transcripts and similarly brighter the red, more enriched are the transcripts. Wherever log$_2$[fold change] is > +1.5 and <—1.5, the corresponding p_{adj} is <0.001. Wherever the fold change is less prominent but significant, the corresponding p_{adj} is often <0.05. Non-significant changes are represented by white blanks without values.

Fig. S7.

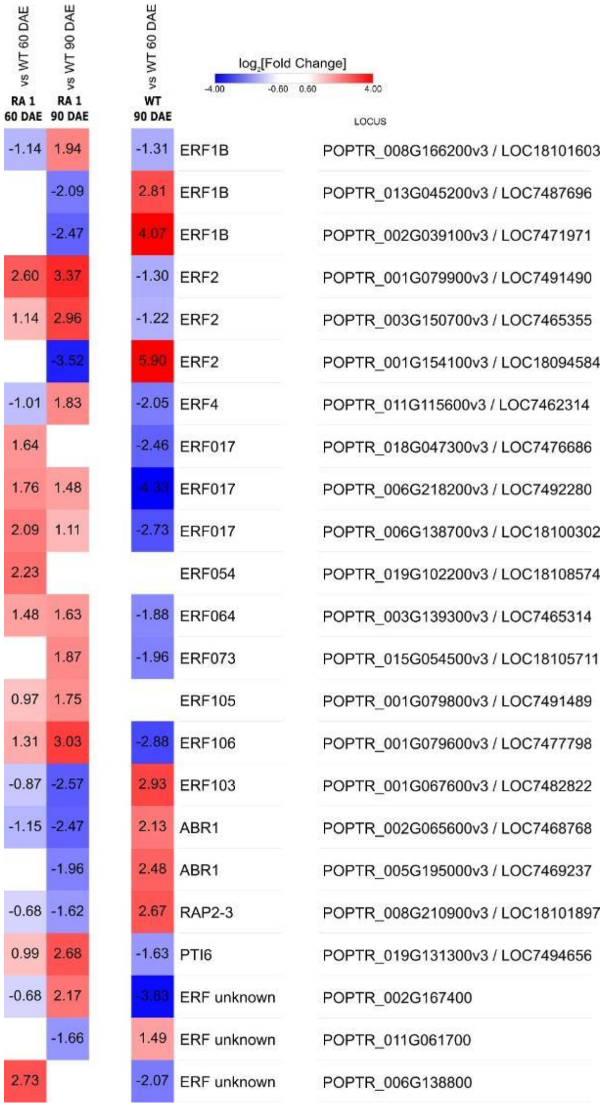

Fig. S7: Impact of isoprene-suppression in poplar leaves on Ethylene Response Factors (ERFs) implicated in regulation of plant stress response pathways. Column descriptions are as given in Fig. S5. Darker the blue, more depleted are the transcripts and similarly brighter the red, more enriched are the transcripts. Wherever $\log_2$[fold change] is > +1.5 and <−1.5, the corresponding p_{adj} is <0.001. Wherever the fold change is less prominent but significant, the corresponding p_{adj} is often <0.05. Non-significant changes are represented by white blanks without values.

Fig. S8

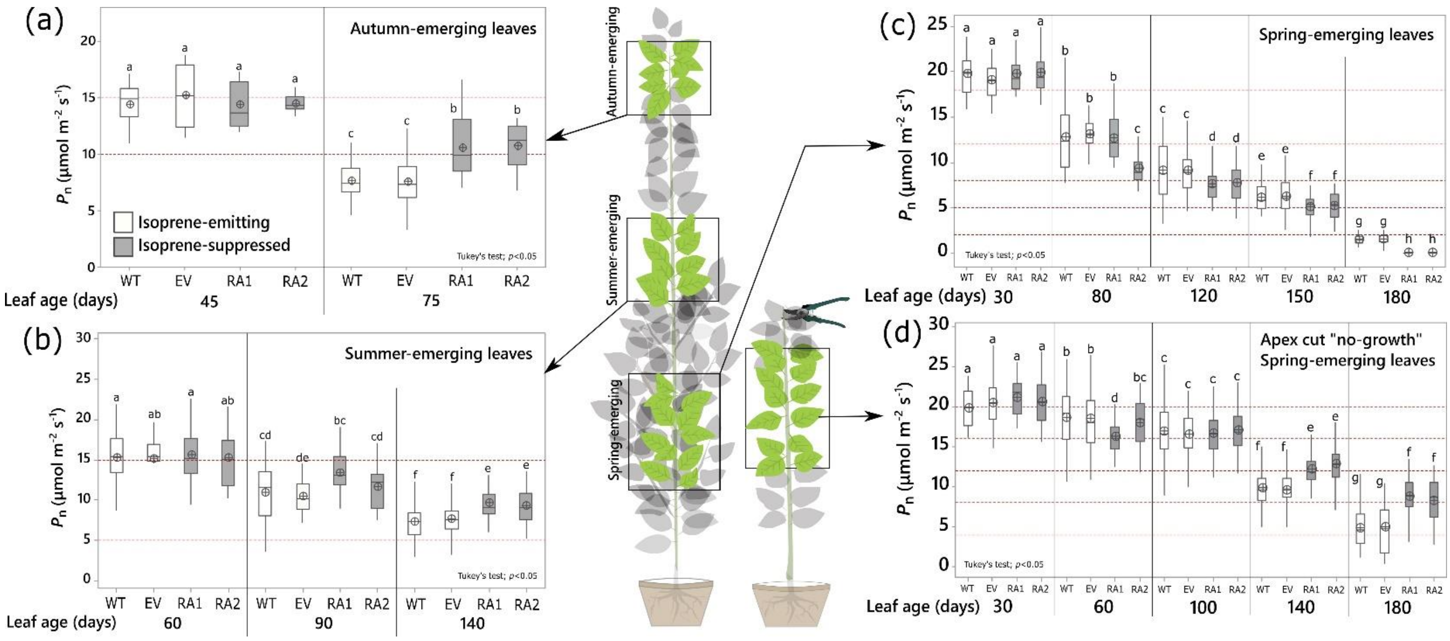

Fig. S8: Contrast between age-specific decline in net photosynthesis (P_n) in apically intact and "no growth" poplars. (a) autumn-emerging leaves (b) summer-emerging leaves (for year 2, compare year 1 data given in main Fig. 1G), (c) spring-emerging leaves of apically intact isoprene-emitting (WT and EV; white bars) and isoprene-suppressed (RA1 and RA2; grey bars) poplars. (d) Leaf age-specific decline in P_n in spring-emerging leaves in poplars subjected to "imposed no-growth". (All data from multiple leaves sampled from $N \geq 5$ biological replicates per line; Tukey' test; α=0.05)

Fig. S9

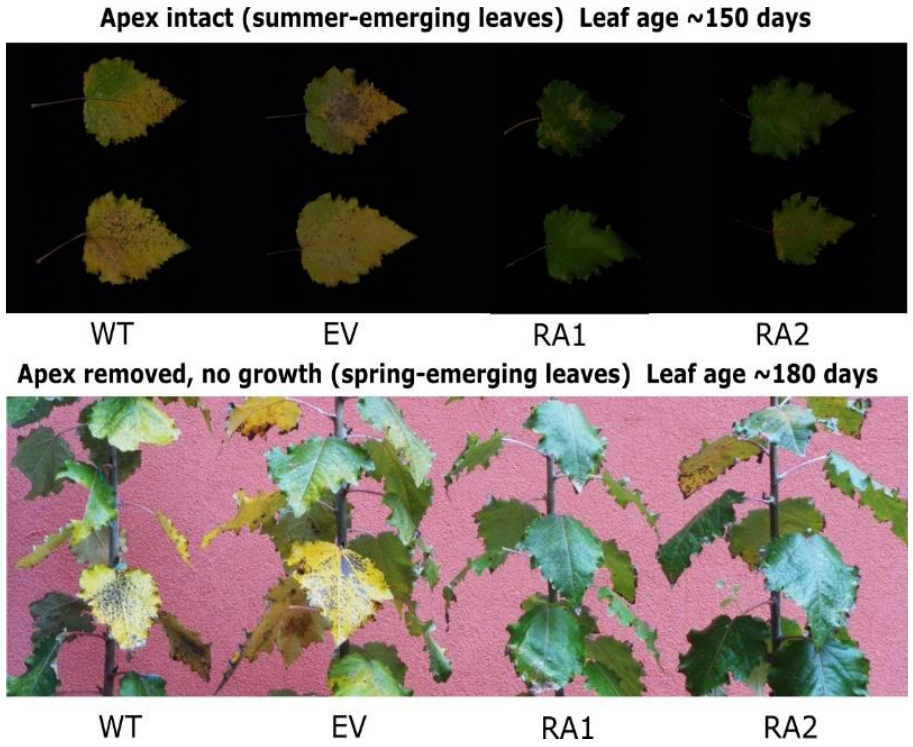

Fig. S9: Photographs showing accelerated leaf senescence in isoprene-emitting (WT and EV) poplars in apically intact poplar (top panel) and the same preserved even under “imposed no-growth” condition (bottom panel). See methods for details.

Table S1

**Table S1.** Parameters of LC-MS/MS analysis of cytokinins in positive mode (see methods for details)							
**Compound**	**Q1 (m/z)**	**Q3 (m/z)**	**Retention Time (min)**	**DP**	**CE**	**CXP**	**Internal standard**
trans-zeatin (tZ)	220.2	136.3	2.4	40	25	16	$[^2H_5]$ tZ
trans-zeatin-riboside (tZR)	352.2	220.3	3.4	40	25	30	$[^2H_5]$ tZR
trans-zeatin-O-glucoside (tZOG)	382.1	220.2	2.3	40	29	18	$[^2H_5]$ tZOG
cis zeatin-riboside (cZR)	352.2	220.3	3.6	40	25	30	$[^2H_5]$ tZR
isopentenyladenine riboside (iPR)	336.1	204.3	5.0	40	23	26	$[^2H_6]$ iPR
isopentenyl adenine (iP)	204.1	136.0	4.1	40	23	16	$[^2H_6]$ iP
DP, declustering potential; CE, collision energy; CXP, collision cell exit potential							

Table S2 and associated supplementary notes

Table S2. Abundance of isopentenyladenine riboside (iPR) and cis zeatin-riboside (cZR) in *Arabidopsis* leaves sampled before flowering respectively for each line. WT and EV-B3 are non-emitting controls. ISPS-B2 and ISPS-C4 are isoprene-emitting lines. Plant age is given as Days after germination (DAG) in parenthesis. Kruskal Wallis *H* test for equality of medians is applied. Each biological replicate (N) includes fully expanded leaves sampled and pooled from at least 4 individuals.

**CK species (ng g FW^{-1})**	**Factor**	**WT (56 DAG)**	**EV-B3 (56 DAG)**	**ISPS-B2 (52 DAG)**	**ISPS-C4 (48 DAG)**	**Factor DOF**	**H-value**	***p***
iPR	N	5	4	4	4	3	10.4	<0.05
	Median	0.41	0.41	0.59	9.68			
	Mean rank	6	5.5	9.8	15.5			
	z-value	-1.58	-1.59	0.34	2.94			
cZR	N	5	4	4	4	3	13.33	<0.05
	Median	0.21	0.14	0.36	27.11			
	Mean rank	8.6	2.5	9.5	15.5			
	z-value	-0.21	-2.94	0.23	2.94			

Notes S1
Supplementary note associated with Table S2

Exceptionally high iPR at 48 DAG, just before flowering, only in isoprene-emitting *Arabidopsis* lines (particularly in ISPS-C4 as seen in Table S2 below) could be an ephemeral phenomenon, which occurs when flowering is artificially induced through photoperiodic intervention (Bernier *et al.,* 1993; Corbesier *et al.,* 2003), except that photoperiodic perturbation did not induce flowering in these plants. This unusually high level of CK-ribosides was not caught in non-emitting lines prior to they started flowering later (56 DAG).

Table S3 and associated supplementary notes

Table S3: Above-ground phenotype of apically intact poplars calculated at the time of final harvest after first year's monitoring of growth and senescence. Trait values represent mean ± SE for 4 biological replicates sampled except plant height (where N =10 individuals). Significant differences are marked by different letters of the alphabet in superscripts next to each value (Tukey's test, $\alpha = 0.05$).

**Trait**	**WT**	**EV**	**RA1**	**RA2**	***p***
Plant height (cm)	240 ± 4 [a]	237 ± 4 [a]	**186 ± 5 [c]**	**211 ± 4 [b]**	<0.05
Average length of sylleptic branches (cm)	43 ± 3	44 ± 3	47 ± 1	49 ± 3	NS
Average total no. of branches per individual (count)	22 ± 2	19 ± 1	22 ± 3	20 ± 2	NS
Average number of branches per 1 m height	9 ± 1	8 ± 1	11 ± 1	9 ± 1	NS
Average length of sylleptic branches (cm) adjacent to nodal spring-emerging leaves. Bottom-most 12 branches were measured per individual.	36 ± 2 [a]	36 ± 2 [a]	**44 ± 3 [a]**	**48 ± 2 [a]**	NS
Average cumulative leaf fresh weight on sylleptic branches (g)	110 ± 6 [bc]	107 ± 9 [c]	**141 ± 7 [a]**	**138 ± 7 [ab]**	<0.05

Notes S2

Supplementary note associated with Table S3

Plant height (length of the main stem or leader), length of sylleptic branches, total number of sylleptic branches, and average number of branches per unit plant height for poplars are tabulated in supplementary table S3. Total number of branches and the number of branches per unit height did not differ among poplar lines, despite significant difference in height, indicating internodal elongation in IE poplars. The lower-most branches were significantly longer and the leaf biomass on branches (cumulative) was significantly greater in isoprene non-emitting poplars (NE: RA1 and RA2) than in isoprene-emitting poplars (IE: WT and EV) poplars. In addition, the cumulative leaf biomass on branches was significantly higher in NE poplars. All provided a direct measure of bushiness in NE poplars compared to apically dominant IE poplars (Fig. 1f).

There was no difference among lines in the timing of bud-break (mid-March). Net photosynthesis (P_n) in summer-leaves did not differ among lines up to 60 DAE, and P_n of spring-leaves did not differ among lines in young leaves (30 DAE). However, at 90 DAE and 140 DAE, P_n decreased more in IE summer-leaves than in NE summer-leaves and the same trend was observed even in relatively younger fully-expanded autumn-leaves just below the shoot apex. Summer-leaves were shorter lived (160 ±10 days) than spring-leaves (180 ± 10 days), in all apically intact poplars. This was potentially due to the fact that summer-leaves were presumably acting as nutrient source to the apical branch which were longer than all other sylleptic (side) branches in all poplar lines. However, the sylleptic branches were much longer in NE poplars than apically stronger IE poplars, which may have contributed to faster senescence of spring-leaves in NE poplars captured by a steeper decline in P_n of NE spring-leaves than in IE spring-leaves during senescence (contrast with the trends in summer-leaves and autumn-leaves, Fig S8a and b). In decapitated "no-growth" poplars, spring-leaves senesced significantly later than their counterparts in all apically intact poplars (both IE and NE lines). Notably, P_n declined significantly more and sooner in decapitated IE leaves than in decapitated NE poplar leaves (140 DAE and 180 DAE, Fig. S8d above). This is similar to the trend observed in the summer-leaves of apically intact plants (Fig. S9; also see main Fig. 1f). While the modular nature of the tree body with its contiguous sources and sinks (Sprugel *et al.*, 1991) had some role in causing the contrasting senescence course of spring- and summer-leaves of all apically intact poplars, when the growth was taken out of the equation, isoprene-emitting leaves senesced earlier than their non-emitting counterparts, irrespective the time of emergence and relative position on the main plant stem. The fact the both IE and NE poplars accrue equivalent total biomass in a growing season (Monson *et al.*, 2020; also this study), despite showing distinct phenotypes (this study) is further proof that new growth is only one among many equally important and intrinsic leaf-specific constraints on the timing and pace of leaf senescence.

Supporting Information references

Bernier G, Havelange A, Houssa C, Petitjean A, Lejeune P. 1993. Physiological signals that induce flowering. *The Plant Cell*, 5, 1147-1155.

Corbesier L, Prinsen E, Jacqmard A, Lejeune P, Van Onckelen H, Périlleux C, Bernier G. 2003. Cytokinin levels in leaves, leaf exudate and shoot apical meristem of Arabidopsis thaliana during floral transition. *Journal of Experimental Botany*, 54, 2511-2517.

Monson RK, Winkler B, Rosenstiel TN, Block K, Merl-Pham J, Strauss SH, Schnitzler JP. 2020. High productivity in hybrid-poplar plantations without isoprene emission to the atmosphere. *Proceedings of National Academy of Sciences USA* 117, 1596-1605.

Sprugel DG, Hinckley TM, Schaap W. 1991. The theory and practice of branch autonomy. *Annual Review of Ecology and Systematics.* 22, 309-334.
